# CircRNA-vgll3 promotes osteogenic differentiation of adipose-derived mesenchymal stem cells via modulating miRNA-dependent integrin α5 expression

**DOI:** 10.1038/s41418-020-0600-6

**Published:** 2020-08-19

**Authors:** Dandan Zhang, Ni Ni, Yuyao Wang, Zhimin Tang, Huiqin Gao, Yahan Ju, Na Sun, Xiaoyu He, Ping Gu, Xianqun Fan

**Affiliations:** Department of Ophthalmology, Ninth People’s Hospital, Shanghai Jiaotong University School of Medicine, Shanghai Key Laboratory of Orbital Diseases and Ocular Oncology, Shanghai, 200011 P.R. China

**Keywords:** Epigenetics, Stem-cell research

## Abstract

Adipose-derived mesenchymal stem cells (ADSCs) are promising candidate for regenerative medicine to repair non-healing bone defects due to their high and easy availability. However, the limited osteogenic differentiation potential greatly hinders the clinical application of ADSCs in bone repair. Accumulating evidences demonstrate that circular RNAs (circRNAs) are involved in stem/progenitor cell fate determination, but their specific role in stem/progenitor cell osteogenesis, remains mostly undescribed. Here, we show that circRNA-vgll3 originating from the vgll3 locus markedly enhances osteogenic differentiation of ADSCs; nevertheless, silencing of circRNA-vgll3 dramatically attenuates ADSC osteogenesis. Furthermore, we validate that circRNA-vgll3 functions in ADSC osteogenesis through a circRNA-vgll3/miR-326-5p/integrin α5 (Itga5) pathway. Itga5 promotes ADSC osteogenic differentiation and miR-326-5p suppresses Itga5 translation. CircRNA-vgll3 directly sequesters miR-326-5p in the cytoplasm and inhibits its activity to promote osteogenic differentiation. Moreover, the therapeutic potential of circRNA-vgll3-modified ADSCs with calcium phosphate cement (CPC) scaffolds was systematically evaluated in a critical-sized defect model in rats. Our results demonstrate that circRNA-vgll3 markedly enhances new bone formation with upregulated bone mineral density, bone volume/tissue volume, trabeculae number, and increased new bone generation. This study reveals the important role of circRNA-vgll3 during new bone biogenesis. Thus, circRNA-vgll3 engineered ADSCs may be effective potential therapeutic targets for bone regenerative medicine.

## Introduction

Mesenchymal stem cells (MSCs) including adipose-derived mesenchymal stem cells (ADSCs) and bone marrow stem cells (BMSCs) are multipotent cells originating from the mesoderm of early development and possess the capacity of self-renewal and differentiation into osteocytes, chondrocytes and adipocytes [1]. MSCs, usually combined with biomedical materials, hold great promise for regenerative medicine, especially for treating the unmet critical-sized bone defects in clinics, which resulted from infection, trauma, osteoporosis, and many systemic abnormalities [2]. Compared to BMSCs, ADSCs, which are located in the stromal-vascular section of adipose tissue, gradually come into focus of choice in bone regenerative medicine due to their abundant and easy autologous origin along with osteogenic capacity [3, 4]. However, the limited osteogenic differentiation potential and a native tendency to differentiate into adipogenic cells greatly hindered the clinical application of ADSCs in bone repair [4]. ADSC osteogenesis involves regulation of multiple factors including noncoding RNAs, while the intracellular molecular regulatory network remains complicated and largely uncharacterized [5, 6]. Therefore, the purpose of this study was to uncover the modulatory factors in osteogenic differentiation of ADSCs and lay a foundation for further application of ADSCs in bone regeneration.

Circular RNAs (circRNAs) are newly concerned RNAs which can form closed continuous rings covalently [7]. They are conserved between species with specific expression in different tissues and developmental stages [8]. CircRNAs are more stable than linear RNAs because they lack a free end for RNA enzyme-mediated degradation [9]. In addition, a series of circRNAs have been verified to undertake pivotal roles in biological activities relating nervous system diseases, cancer as well as the development, controlled pluripotency, and regulated differentiation of stem cells [10–13]. As such, circRNAs are expected to serve as new diagnostic and treatment strategies for clinical medicine. Among them, the role of circRNAs in osteogenesis begun to attract attention. Some researchers have reported changes of circRNAs in osteogenesis by high-throughput sequencing and prediction [14, 15], suggesting the possible roles of circRNAs in osteogenesis. Thus, the specific roles of circRNAs in osteogenesis as well as the related underlying mechanisms need further exploration.

Concerning the mechanisms of action for circRNAs, one mode reported was the microRNAs (miRNAs) “sponges” activity of some exonic circRNAs [16]. CircRNAs may perform their function through miRNA “sponges” to influence the expression of downstream target genes, which is validated in various biological activities [17, 18]. MiRNAs play important roles in tissue development by forming complete or partial complementarity with 3′ noncoding region (3′ UTR) of the target genes’ mRNA [19, 20]. Groups worldwide have reported the essential role of miRNAs mediating the maintenance of MSC identity or modulation of osteogenesis, including miR-214, miR-34, miR-133, miR-210 and miR-125b [21–25]. Our group has also found miRNAs including miR-31, miR-135, miR-26a, miR-146a that are involved in the osteogenesis of MSCs [26–29]. At present, with the gradual acquaint with circRNAs, the relationships of circRNAs, miRNAs and osteogenesis remain mostly undescribed.

The positive roles of integrins in osteogenesis have been documented [30, 31]. Integrins including subtype Integrin α5 (Itga5) have essential roles in cell attachment to the extracellular matrix (ECM) and osteoprogenitor survival and function [32]. It was reported that many factors that regulate bone formation including bone morphogenetic protein 2 (BMP2), transforming growth factor beta (TGFβ), parathyroid hormone (PTH) and mechanical stress function partly through regulating the expression of Itga5 [33–36]. Thus, novel integrin-targeted approaches can enhance the osteoprogenitor cell homing, recruitment and osteogenic differentiation, which can be translated into increased bone formation [32].

In this study, we explored the functional roles and underlying mechanisms of circRNAs in regulating ADSC osteogenic differentiation for the first time. Exon-derived circRNA-vgll3 played a positive role in modulating ADSC osteogenic differentiation. Additionally, circRNA-vgll3 can competitively bind miR-326-5p, which in turn reversely regulated the translation of the target gene Itga5, thereby regulating ADSC osteogenic differentiation activity. Besides, the effects of combination of circRNA-vgll3-modified ADSCs with synthetic calcium phosphate cement (CPC) scaffolds, which are acknowledged biocompatible hard tissue biomedical materials [37], for repairing critical-sized bone defects in vivo were explored further. Our data indicate that circRNA-vgll3-engineered ADSCs are highly promising for enhancing bone regeneration and facilitating cure of critical-sized bone defects in the future.

## Results

### Characterization of circRNA-vgll3 in ADSCs

**Fig. 1:**
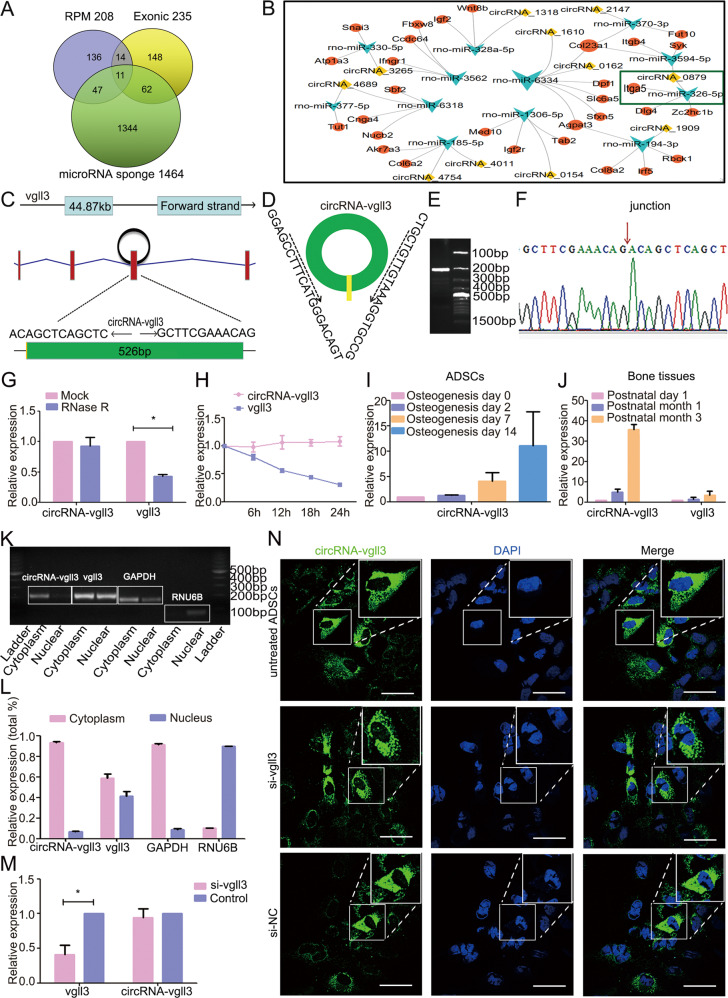
Characterization of circRNA-vgll3 in ADSCs. **a** Venn analysis of abundant (RPM > 0.1) exonic circRNAs with potential miRNA binding sites in ADSCs. **b** Bioinformatics prediction of 11 circRNAs, their predicted miRNAs, and miRNA targets. **c** CircRNA-vgll3 generated from the third exon of vgll3 gene locus. **d**-**f** PCR validation of circRNA-vgll3 using outward-facing primer and Sanger sequencing of the PCR product. **g** The relative gene expression of circRNA-vgll3 and vgll3 after RNase R treatment (*n* = 3, *P < 0.05 versus Mock group, statistical analysis was performed by Student’s *t* test). **h** The relative gene expression changes of circRNA-vgll3 and vgll3 after actinomycin D treatment for 6 h, 12 h,18 h, and 24 h, compared to that of 0 h (*n* = 3). **i** qPCR analysis of circRNA-vgll3 expression in BMP2-induced ADSCs and naive ADSCs. **j** qPCR analysis of circRNA-vgll3 expression in bone tissue development time points of postnatal day 1, month 1 and month 3. **k**-**l** PCR and qPCR analysis of cytoplasmic and nuclear RNA with circRNA-vgll3 primer, vgll3 primer, GAPDH primer (canonical marker of cytoplasmic fraction) and RNU6B primer (canonical marker of nuclear fraction) showed that circRNA-vgll3 mainly located in the cytoplasm and vgll3 located in the cytoplasm and nucleus. **m** qPCR analysis of the efficiency of the si-vgll3 in knocking down the mRNA levels of vgll3 (*n* = 3, *P < 0.05 versus Control group, statistical analysis was performed by Student’s *t* test). **n** FISH results depicted the cytoplasm location of circRNA-vgll3. Si-vgll3 treatment did not alter the labeling efficiency. Scale bars: 60 µm.

ADSCs were identified by flow cytometry and showed high expression of CD29 and CD90 and hardly express CD45 and CD31 (Supplementary Fig. 1). CircRNA transcripts were characterized by ribosomal RNA-depleted total RNA sequencing analyses in BMP2-induced ADSCs and naive ADSCs. CIRI software, which did not rely on gene annotations [38], was used for identifying circRNAs. Totally, 235 distinct circRNA candidates were predicted to be exonic circRNAs (Fig. 1a). Highly expressed circRNAs were classified as RPM > 0.1 (RPM, the percentage of mapped back-splice junction reads in million mapped reads) [39], and 208 circRNAs match this condition (Fig. 1a). We used miRanda (www.microrna.org) software to predict miRNA–circRNA interactions to help resolve the functions and mechanisms of the circRNAs, showing that 1464 circRNAs share miRNA binding sites (Fig. 1a). Only 11 circRNAs meet the above three simultaneous requirements (exonic, abundant and have a miRNA–circRNA interaction) (Fig. 1a, Supplementary Fig. 2). The miRNA–circRNA interactions of the 11 circRNAs were shown in Fig. 1b.

CircRNA-0879 (termed circRNA-vgll3 in this study) is originated from the third exon of the vgll3 gene (Fig. 1c), which used to be reported to determine the osteogenic and adipogenic differentiation of MSCs [40, 41]. Thus, circRNA-vgll3 was chosen for further study. Using outward-facing primers, distinct products which were consistent with the expected sizes were amplified and further confirmed by Sanger sequencing (Fig. 1d–f). Quantitative polymerase chain reaction (qPCR) results displayed that circRNA-vgll3 was resistant to RNase R, a known exoribonuclease, and actinomycin D, a known RNA polymerase inhibitor (Fig. 1g, h). The stable property supports that circRNA-vgll3 has a circular structure. Moreover, the expression levels of circRNA-vgll3 were gradually upregulated during the BMP2-induced ADSC osteogenic differentiation (Fig. 1i) and also obviously upregulated in the development of bone tissues (Fig. 1j) as indicated by qPCR. CircRNA-vgll3 was more inclined to localize in the cytoplasm as evidenced by the nucleoplasmic separation experiments (Fig. 1k, l). Besides, after knocking down the mRNA levels of vgll3 by si-vgll3 (Supplementary Fig. 3, Fig. 1m), specific probe was designed in the backsplicing junction of circRNA-vgll3 to label the circRNAs. RNA fluorescence in situ hybridization (FISH) results demonstrated the cytoplasm location of circRNA-vgll3 in untreated ADSCs. The non-influenced positive labeling in si-vgll3 treated ADSCs suggested that the probe was specific to circRNA-vgll3 and did not label the vgll3 mRNA (Fig. 1n). Taken together, our data showed that circRNA-vgll3 was a stable and abundant circRNA expressed in ADSCs, and the endogenous expression levels were gradually upregulated during ADSC osteogenic differentiation.

### CircRNA-vgll3 positively regulates ADSC osteogenesis

**Fig. 2:**
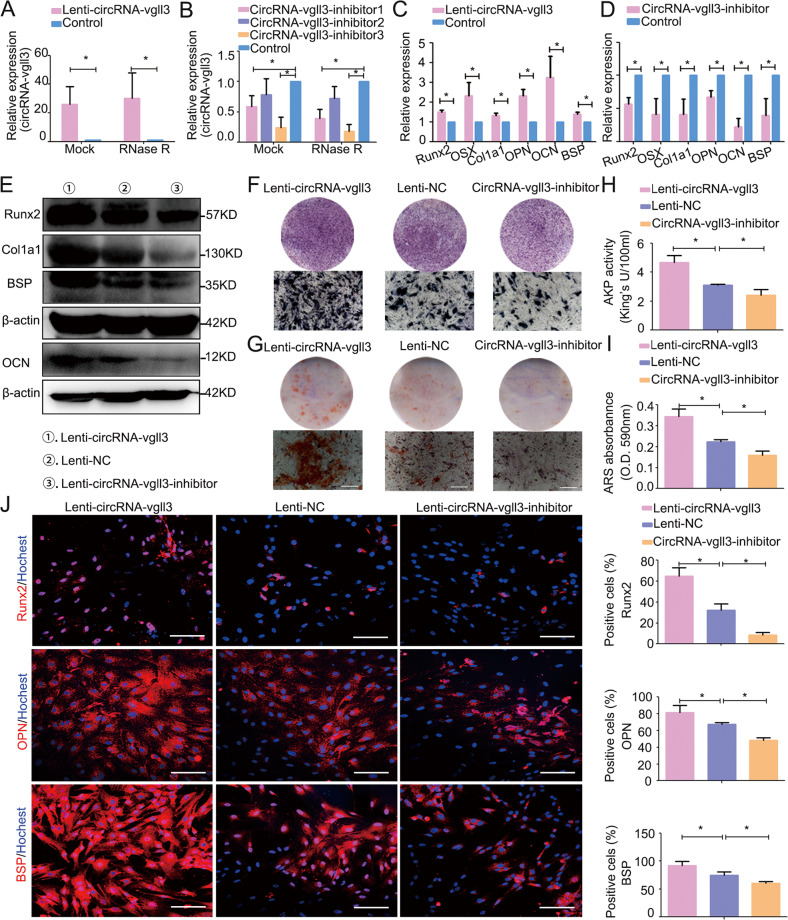
CircRNA-vgll3 positively regulates ADSC osteogenesis. **a** Transfection efficiency was evaluated by qPCR and showed upregulation of circRNA-vgll3 expression in the Lenti-circRNA-vgll3-transfected group. The transfection efficiency was not influenced by RNase R treatment (*n* = 3, *P < 0.05 versus Control group, statistical analysis was performed by Student’s *t* test). **b** Three shRNAs for circRNA-vgll3 were cloned into vectors, and qPCR analysis showed that circRNA-vgll3 inhibitor 1 and 3 can effectively knockdown the expression of circRNA-vgll3. The knockdown efficiency was not influenced by RNase R treatment (*n* = 3, *P < 0.05 versus Control group, statistical analysis was performed by one-way ANOVA). **c**–**d** mRNA expression levels of the markers Runx2, OSX, Col1a1, OPN, OCN, and BSP were elevated at osteogenesis day 7 by Lenti-circRNA-vgll3 whereas they were impaired by circRNA-vgll3 inhibitor when compared to Lenti-NC (*n* = 3, *P < 0.05 versus Control group, statistical analysis was performed by Student’s *t* test). **e** Western blot analysis detecting the levels of Runx2, Col1a1, BSP and OCN at osteogenesis day 7 showed that they were significantly promoted by Lenti-circRNA-vgll3 and greatly attenuated by transfection of the circRNA-vgll3 inhibitor in comparison to Lenti-NC. **f** ALP staining evaluated the effect of circRNA-vgll3 on ALP activity at osteogenesis day 7. Scale bars: 100 µm. **g** ARS staining evaluated the effect of circRNA-vgll3 on the ECM mineralization at osteogenesis day 14. Scale bars: 100 µm. **h**–**i** Semiquantitative analysis of ALP activity and ARS activity (*n* = 3, *P < 0.05 versus Control group, statistical analysis was performed by one-way ANOVA). **j** Cellular immunofluorescence showing the cellular expression levels of Runx2, OPN and BSP at osteogenesis day 7 (*n* = 3, *P < 0.05 versus Control group, statistical analysis was performed by one-way ANOVA, Scale bars: 100 µm).

The Lenti-circRNA-vgll3, circRNA-vgll3 inhibitor and Lenti-NC were transfected into ADSCs to investigate the regulatory role of circRNA-vgll3 on BMP2-induced ADSC osteogenesis. The MOI selection for ADSCs shown in Supplementary Fig. 4 depicted that in the MOI = 20 group, the positive ratio of green fluorescence-positive cells reached more than 80%. Thus, MOI = 20 was chosen for further experiments. We detected the tag sequence of ZsGreen and puro of the lenti-virus in the ADSC genomics using the Next Generation Sequencing (NGS) technology, and consequently, we detected the presence of ZsGreen and puro in the genomics (chrX: 77262913), indicating the successful genomic integration of lenti-virus (Supplementary Fig. 5). Transfection efficiency results showed approximately 26-fold upregulation of circRNA-vgll3 expression in the Lenti-circRNA-vgll3-transfected group and confirmed a resistant to RNase R digestion of the over-expressed circRNA-vgll3, indicating the overexpression was specific to circRNA-vgll3 (Fig. 2a). Three shRNAs for circRNA-vgll3 inhibited the expression of circRNA-vgll3 (0.589-fold, 0.78-fold, and 0.245-fold for circRNA-vgll3 inhibitors 1, 2, and 3, respectively) in ADSCs while did not affect the expression of the vgll3 mRNA (Fig. 2b, Supplementary Fig. 6). In addition, in order to demonstrate that the knockdown is specifically to circRNA-vgll3, we used RNase R to digest RNA, and our results showed a consistent knockdown by circRNA-vgll3 inhibitors, indicating that the knockdown is specially to the circRNA-vgll3 (Fig. 2b). CircRNA-vgll3 inhibitor 3 was chosen for further studies and is referred to as circRNA-vgll3 inhibitor.

The mRNA expression levels of osteogenic marker genes in ADSCs including Runt-related transcription factor 2 (Runx2), osterix (OSX), collagen1 a1 (Col1a1), osteopontin (OPN), osteocalcin (OCN) and bone sialoprotein (BSP) were markedly enhanced by Lenti-circRNA-vgll3 and suppressed by circRNA-vgll3 inhibitor comparing to Lenti-NC (Fig. 2c, d). The protein expression levels of Runx2, Col1a1, OCN and BSP detected by western blot analysis demonstrated a consistent trend (Fig. 2e). ALP staining (Fig. 2f) and ARS staining (Fig. 2g) results demonstrated that Lenti-circRNA-vgll3 obviously promoted ALP activity and calcium deposition, whereas the circRNA-vgll3 inhibitor impaired these processes. Semiquantitative analysis of ALP and ARS activity revealed a similar pattern (Fig. 2h, i). Moreover, cellular immunofluorescence results showed that the ratios of Runx2-, OPN- and BSP-immune-positive cells were much higher (64.9 ± 8.0%, 81.6 ± 8.2%, and 92.0 ± 7.2%, respectively) (*P* < 0.05) in Lenti-circRNA-vgll3-transfected ADSCs, but were obviously lower in circRNA-vgll3 inhibitor-transfected ADSCs comparing to Lenti-NC (Fig. 2j). Thus, our data suggest that circRNA-vgll3 enhanced the osteogenic differentiation of ADSCs.

### MiR-326-5p regulates ADSC osteogenesis by targeting Itga5

**Fig. 3:**
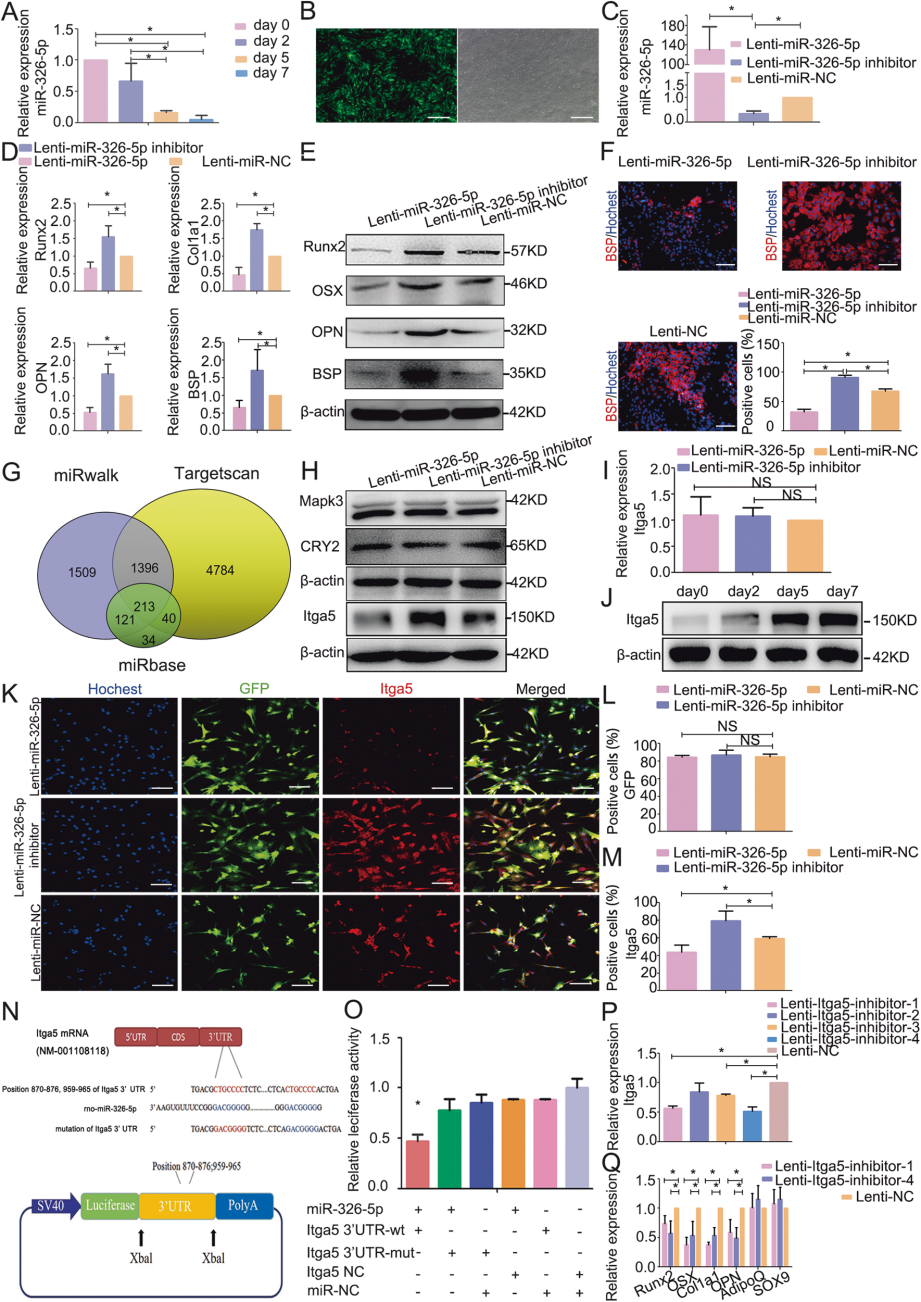
MiR-326-5p regulates ADSC osteogenesis by targeting Itga5. **a** MiR-326-5p expression levels during ADSC osteogenic differentiation were assessed by qPCR and showed a gradual trend of downregulation (*n* = 3, *P < 0.05, statistical analysis was performed by one-way ANOVA). **b** Transfection efficiency of miR-326-5p as evidenced by fluorescence pictures. Scale bars: 200 µm. **c** Transfection efficiency of Lenti-miR-326-5p and Lenti-miR-326-5p inhibitor was evaluated by qPCR (*n* = 3, *P < 0.05 versus Lenti-miR-NC group, statistical analysis was performed by one-way ANOVA). **d** The mRNA expression of osteogenic marker genes Runx2, Col1a1, OPN and BSP was markedly inhibited in the Lenti-miR-326-5p-transfected cells whereas it was promoted by the Lenti-miR-326-5p inhibitor compared to Lenti-NC at osteogenesis day 7 (*n* = 3, *P < 0.05 versus Lenti-miR-NC group, statistical analysis was performed by one-way ANOVA). **(e)**. Western blotting for the protein expression of Runx2, OSX, OPN and BSP at osteogenesis day 7. **f** Immunocytochemistry showed BSP was markedly inhibited in the Lenti-miR-326-5p-transfected cells whereas it was promoted by the Lenti-miR-326-5p inhibitor compared to Lenti-NC. (*n* = 3, *P < 0.05, statistical analysis was performed by one-way ANOVA, Scale bars: 100 µm). **g** The target gene prediction databases miRanda, miRwalk and Targetscan indicate that there are totally 213 target genes in intersection for miR-326-5p. **h** The protein levels of CRY2 and Mapk3 were not influenced by overexpression or knockdown of miR-326-5p in ADSCs. The protein level of Itga5 was reduced in Lenti-miR-326-5p-transfected ADSCs whereas it was promoted in Lenti-miR-326-5p inhibitor-transfected ADSCs at osteogenesis day 7. **i** qPCR results of Itga5 mRNA expression in Lenti-miR-326-5p-transfected ADSCs at osteogenesis day 7. **j** Western blot analysis at days 0, 2, 5, and 7 indicated that the protein expression of Itga5 was gradually increased. **k**–**m**. Immunocytochemistry analysis of Itga5 expression levels in Lenti-miR-326-5p-transduced ADSCs. Scale bars: 100 µm. **n** Schematic diagram depicting the constructed firefly luciferase reporter system and the predicted binding sites of miR-326-5p in the Itga5 3′UTR. **o** Luciferase reporter assay showed that co-transfection of miR-326-5p with the constructed Itga5 3'UTR-wt plasmid obviously decreased luciferase activity (*P < 0.05, statistical analysis was performed by one-way ANOVA). **p** Four Lenti-itga5-inhibitors were transduced into ADSCs, and Lenti-itga5-inhibitor-1 and Lenti-itga5-inhibitor-4 effectively knocked down the expression of Itga5 (*n* = 3, *P < 0.05 versus Lenti-NC group, statistical analysis was performed by one-way ANOVA). **q** Knockdown of Itga5 by Lenti-itga5-inhibitor-1 and Lenti-itga5-inhibitor-4 decreased the mRNA levels of Runx2, OSX, Col1a1 and OPN, while the expression of AdipoQ and SOX9 showed no obvious changes with Itga5 inhibition in ADSCs at osteogenesis day 7 (*n* = 3, *P < 0.05 versus Lenti-NC group, statistical analysis was performed by one-way ANOVA).

Given that circRNA-vgll3 is abundant in the cytoplasm, the ability of circRNA-vgll3 to bind miRNAs was next investigated. MiRanda software predicted that circRNA-vgll3 has 2 potent binding sites for miR-326-5p (Supplementary Fig. 7). qPCR assessing the levels of miR-326-5p showed a gradual trend of downregulation during BMP2-induced ADSC osteogenic differentiation (Fig. 3a). The transfection efficiency of Lenti-miR-326-5p and Lenti-miR-326-5p inhibitor was evaluated by fluorescence detection and qPCR analysis, showing approximately 130.84-fold upregulation of miR-326-5p expression in the Lenti-miR-326-5p-transfected group and 0.35-fold miR-326-5p expression in Lenti-miR-326-5p inhibitor-transfected cells (Fig. 3b, c). Osteogenic genes of Runx2, OSX, Col1a1, OPN and BSP expression were obviously inhibited by Lenti-miR-326-5p, whereas were enhanced by Lenti-miR-326-5p inhibitor, as indicated by qPCR or western blotting (Fig. 3d, e) at osteogenesis day 7, consistent with BSP positive ratio (Fig. 3f), suggesting that miR-326-5p negatively modulates ADSC osteogenesis.

The target gene prediction databases miRanda, miRwalk and Targetscan indicate that there are totally 213 target genes for miR-326-5p in intersection (Fig. 3g). However, these predicted targets are not validated in ADSCs before. We analyzed the mRNA sequencing results in BMP2-induced ADSCs and naive ADSCs, and the Kyoto Encyclopedia of Genes and Genomes (KEGG) enrichment analysis results showed that there were several enrich factor ≥ 4 pathways including TGF-β signaling pathway, ECM-receptor interaction, circadian rhythm, sulfer metabolism and other glycan degradation involved in ADSC osteogenesis (Supplementary Fig. 8). Among the 213 genes, only Itga5, cryptochrome circadian regulator 2 (CRY2) and mitogen-activated protein kinase 3 (Mapk3) located in the KEGG mentioned above. After overexpressing or knocking down the expression of miR-326-5p in ADSCs, the protein levels of CRY2 and Mapk3 showed no significant changes at osteogenesis day 7. While the expression of Itga5, which was reported to play essential roles in osteoblast function and survival [32], was reduced in Lenti-miR-326-5p-transfected ADSCs and promoted in Lenti-miR-326-5p inhibitor-transfected ADSCs (Fig. 3h), indicating that Itga5 may be the functional target of miR-326-5p. qPCR analyses at osteogenesis day 7 exhibited no obvious changes in Itga5 mRNA levels after modulation of miR-326-5p levels in ADSCs (Fig. 3i). Western blot analysis of Itga5 protein level on osteogenic days 0, 2, 5, and 7 depicted a time-dependent increasing (Fig. 3j). Immunofluorescence results indicated that the positive ratio of Itga5 in Lenti-miR-NC-transfected ADSCs was 59.17 ± 2.10%. The ratio of Itga5-immunostaining in Lenti-miR-326-5p-transfected cells was distinctively lower (44.10 ± 7.8%), without a difference in green fluorescence-positive cells (Fig. 3k–m). MiRanda database predicted that positions 870–876 and 959–965 in the Itga5 (NM-001108118) 3' UTR are potent binding sites for miR-326-5p (Fig. 3n). To verify this finding, a firefly luciferase reporter plasmid was constructed, which contained either wild type fragments (called Itga5 3' UTR-wt) or mutant binding site fragments (called Itga5 3' UTR-mut) downstream of the firefly sequence (Fig. 3n). The miR-326-5p expression plasmid (referred to as miR-326-5p) or control plasmid (called miR-NC) was co-transfected into 293T cells (Fig. 3o) or ADSCs (Supplementary Fig. 9) with the constructed reporter plasmids. Reporter plasmids co-transfected with miR-NC had no influence on luciferase activity. Compared to Itga5 3' UTR-mut, co-transfection of Itga5 3' UTR-wt with miR-326-5p decreased luciferase activity (Fig. 3o, Supplementary Fig. 9). These data imply that miR-326-5p can post-transcriptionally regulate Itga5 expression.

Additionally, after inhibiting the expression of Itga5, the expression of osteogenic markers Runx2, OSX, Col1a1, and OPN was downregulated, while the expression of adipogenic marker AdipoQ and the chondrogenic marker SOX9 showed no obvious changes, suggesting that Itga5 is a driver for osteogenic differentiation (Fig. 3p, q). Taken together, these data reveal that miR-326-5p negatively regulates ADSC osteogenic differentiation by targeting Itga5.

### CircRNA-vgll3 upregulates the level of Itga5 through miR-326-5p

**Fig. 4:**
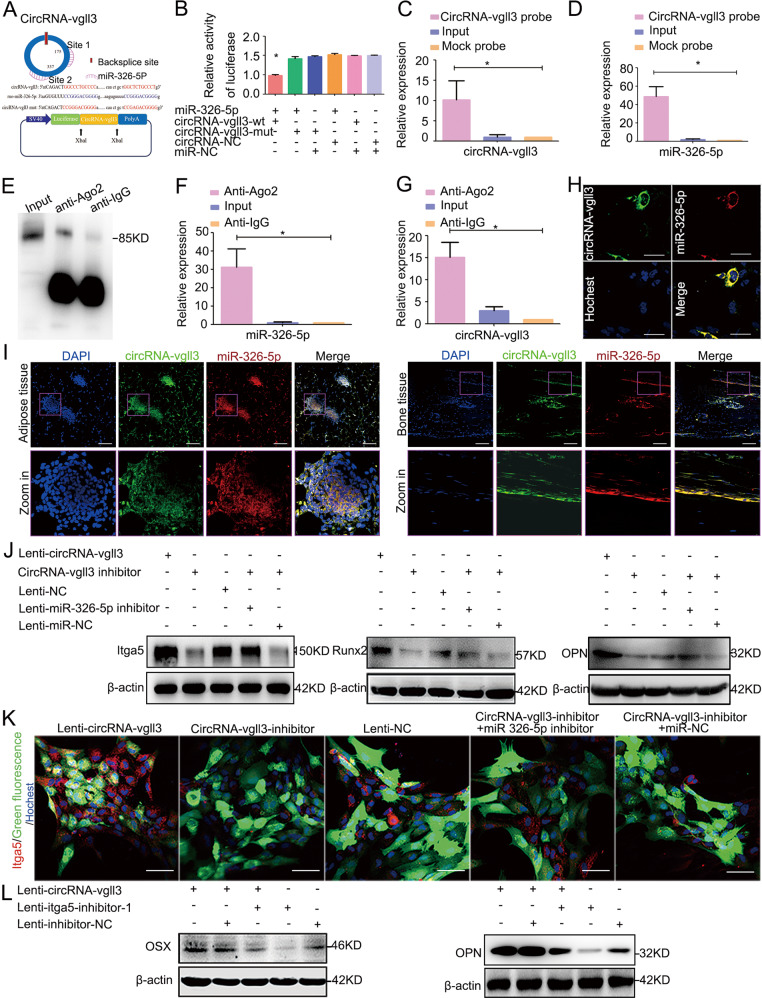
CircRNA-vgll3 upregulates the level of Itga5 through miR-326-5p. **a** The predicted miR-326-5p binding sites on circRNA-vgll3. **b** Luciferase reporter assay showed that co-transfection of the miR-326-5p plasmid with circRNA-vgll3-wt plasmid significantly impaired the luciferase activity (*P < 0.05 versus NC group, statistical analysis was performed by one-way ANOVA). **c**–**d** RNA pull-down analysis showed distinctive enrichment of circRNA-vgll3 and miR-326-5p in the biotin-labeled circRNA-specific probe group (*n* = 3, *P < 0.05 versus Mock probe group, statistical analysis was performed by one-way ANOVA). **e**–**g** RNA RIP was implemented on ADSCs using antibodies against Ago2 to evaluate whether circRNA-vgll3 was related to RISC. Western blot analysis of the efficiency of Ago2 enrichment by anti-Ago2 (**e**); qPCR measuring the RNA levels in immunoprecipitants showed that the miR-326-5p level in the anti-Ago2 group was higher than that in the anti-IgG cells (**f**), accompanied by a higher circRNA-vgll3 level in the anti-Ago2 group than that in the anti-IgG group (**g**) (*n* = 3, *P < 0.05 versus anti-IgG group, statistical analysis was performed by one-way ANOVA)**. h** FISH assay showed both miR-326-5p and circRNA-vgll3 localized in the cytoplasm of ADSCs. Scale bars: 60 µm. **i** FISH assay showed that both miR-326-5p and circRNA-vgll3 localized in the cytoplasm of adipose and bone tissues. Scale bars: 200 µm. **j** ADSCs were co-transfected with the circRNA-vgll3 inhibitor and the miR-326-5p inhibitor, and the expression levels of Itga5, Runx2 and OPN were evaluated by western blotting at osteogenesis day 7. The levels of Itga5, Runx2, and OPN were significantly decreased by transfection with circRNA-vgll3 inhibitor; however, the miR-326-5p inhibitor obviously rescued the low levels of Itga5, Runx2, and OPN caused by the circRNA-vgll3 inhibitor. **k** Immunocytochemistry detection of Itga5 after co-transfection of circRNA-vgll3 inhibitor and the miR-326-5p inhibitor in ADSCs. Scale bars: 50 µm. **l** Co-transfection of Lenti-circRNA-vgll3 and Itga5 inhibitor in ADSCs impaired the upregulated expression of OSX and OPN caused by circRNA-vgll3 overexpression at osteogenesis day 7.

CircRNA-vgll3 was predicted to have two binding sites (binding site 1 and binding site 2) for miR-326-5p (Fig. 4a). To identify whether miR-326-5p binds to circRNA-vgll3, we constructed luciferase reporter plasmids. In the downstream of the luciferase coding region, the fragments in circRNA-vgll3 containing the binding site 1, site 2, and site 1 plus site 2 for miR-326-5p were inserted, which was called circRNA-vgll3-wt 1, circRNA-vgll3-wt 2, and circRNA-vgll3-wt, individually. The miR-326-5p plasmid or miR-NC plasmid was co-transfected with circRNA-vgll3-wt 1 or circRNA-vgll3-wt 2 or circRNA-vgll3-wt into cells (Supplementary Figs. 10,11, Fig. 4b). Compared to miR-NC, miR-326-5p reduced the luciferase reporter activity significantly. We further mutated the predicted miR-326-5p binding site 1, site 2, and site 1 plus site 2 on circRNA-vgll3 (called circRNA-vgll3-mut 1, circRNA-vgll3-mut 2 and circRNA-vgll3-mut), showing co-transfection with miR-326-5p did not significantly alter the luciferase activity (Supplementary Figs. 10,11, Fig. 4b), indicating that both the predicted binding sites 1 and 2 of circRNA-vgll3 can bind miR-326-5p.

To determine the functional miRNAs that may interact with circRNA-vgll3 in ADSCs, a circRNA-specific probe (biotin-labeled probe) was used to perform a RNA pull-down assay. The circRNA-vgll3-associated RNAs were purified using the circRNA-vgll3-specific probe, and the relative enrichment of RNAs was then evaluated in the circRNA-vgll3 probe group and mock group. Compared to the mock group, specific enrichment of circRNA-vgll3 and miR-326-5p was detected in the circRNA-vgll3 probe group (upregulated 10.13- and 48.55-fold, respectively) (Fig. 4c, d), suggesting that miR-326-5p is the circRNA-vgll3-associated miRNA in ADSCs.

Because miRNAs function by binding to Ago2 protein, the core part of the RNA induced silencing complex (RISC), RNA immunoprecipitation (RIP) assay was performed using antibodies to drag Ago2 to investigate whether circRNA-vgll3 associates with RISC. Western blot analysis revealed the efficiency of Ago2 protein enrichment by anti-Ago2 (Fig. 4e). Noticeably, qPCR of the immunoprecipitants exhibited much more enrichment of circRNA-vgll3 in the Ago2 group (upregulated 15.10-fold) compared to the control immunoglobulin G (IgG) group; simultaneously, the miR-326-5p level was much higher in Ago2-containing immunoprecipitants (upregulated 31.258-fold) than that in control IgG immunoprecipitants (Fig. 4f, g). In order to illustrate the level of ‘sponging’, we performed a relative stoichiometries analysis of the Ago2 asscociated miR-326-5p and circRNA-vgll3. Using standards of miR-326-5p and circRNA-vgll3 (the methods are shown in Methods and Materials section, and the amplification plots of the standards and immunoprecipitated samples are shown in Supplementary Fig. 12), we can calculate that there are approximately 1.23×10^7^ copies of miR-326-5p and 4.37 × 10^6^ copies of circRNA-vgll3 captured by Ago2 antibody. Each circRNA-vgll3 has 2 binding sites for miR-326-5p. Thus 4.37 × 10^6^ copies of circRNA-vgll3 can sponge 8.74 × 10^6^ copies of miR-326-5p. In other word, of the total 1.23 × 10^7^ copies of immunoprecipitated miR-326-5p, 8.74 × 10^6^ copies of miR-326-5p are sponged by circRNA-vgll3. Thus, the level of “sponging” is about 71%. In addition, a subsequent FISH assay in ADSCs, adipose tissue and bone tissue manifested that circRNA-vgll3 and miR-326-5p both localized in cytoplasm (Fig. 4h, i). Above all, these evidences imply that circRNA-vgll3 and miR-326-5p are associated in ADSCs.

In order to address whether the RNAs have function for ADSC osteogenic differentiation at endogenous levels, we investigated the relative expression of circRNA-vgll3, miR-326-5p and the osteogenic makers Runx2, OPN by qPCR analyses during the differentiation of ADSCs without transfections. Our data exhibited that in this process, the endogenous levels of osteogenic makers Runx2, OPN were gradually upregulated, accompanied by an upregulated expression of circRNA-vgll3, and a correspondingly gradually downregulated expression of miR-326-5p, suggesting that endogenous levels of circRNA-vgll3 and miR-326-5p may have function in ADSC osteogenic differentiation (Supplementary Fig. 13).

To further address the relationship among circRNA-vgll3, miR-326-5p, and Itga5, co-transfection in ADSCs were applied. Firstly, ADSCs were co-transfected with circRNA-vgll3 inhibitor and miR-326-5p inhibitor. Western blot analysis revealed that circRNA-vgll3 inhibitor obviously inhibited the expression of Itga5, Runx2 and OPN; however, when transfected together with miR-326-5p inhibitor, the low expression levels of Itga5, Runx2 and OPN caused by the circRNA-vgll3 inhibitor were rescued (Fig. 4j). Immunocytochemistry of Itga5 depicted a similar trend (Fig. 4k). Further, when ADSCs were co-transfected with Lenti-circRNA-vgll3 and Itga5 inhibitor, the upregulated expression of OSX and OPN caused by circRNA-vgll3 overexpression was impaired (Fig. 4l).

Collectively, the above results indicate that circRNA-vgll3 upregulates the level of Itga5 via miR-326-5p, suggesting their modulatory roles in ADSC osteogenic differentiation.

### CircRNA-vgll3 regulates bone formation in vivo

**Fig. 5:**
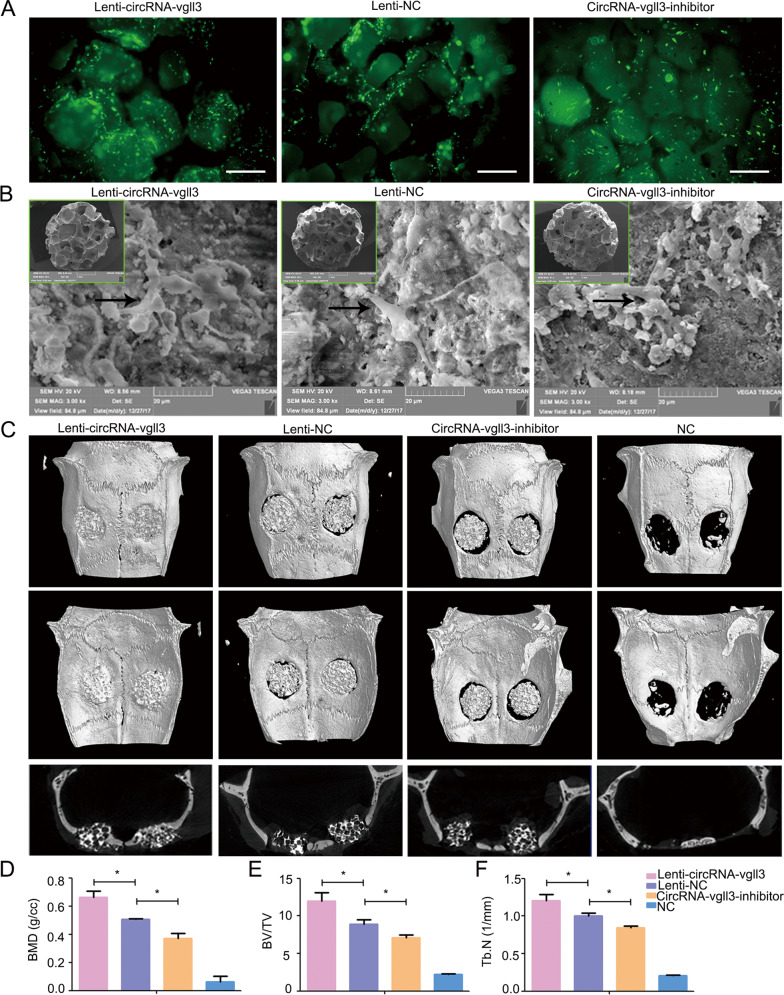
CircRNA-vgll3 regulates bone formation in vivo. **a** Fluorescence images of ADSCs on CPC scaffolds. Scale bars: 200 µm. **b** SEM images revealed that the transfected ADSCs were tightly adherent to the surface and pores of CPCs with a large amount of ECM fibril networks. **c** Micro-CT showed that new bone regeneration was promoted in the Lenti-circRNA-vgll3 group, whereas it was impaired by circRNA-vgll3 inhibitor 8 weeks post implantation. **d**–**f** Micro-CT quantitative analysis showed that the BMD, BV/TV and Tb. N of the overexpression group were markedly enhanced compared to the Lenti-NC group, whereas they were inhibited in the circRNA-vgll3 inhibitor skulls than those in the Lenti-NC skulls. *P < 0.05, statistical analysis was performed by one-way ANOVA.

To establish the therapeutic effects of circRNA-vgll3-engineered ADSCs in healing injured bone, we constructed a critical-sized bone defects model in rat skulls. ADSCs were seeded on CPC scaffolds with Lenti-circRNA-vgll3, circRNA-vgll3 inhibitor or Lenti-NC transfected. Fluorescence images of green fluorescence showed the viability of transfected ADSCs on CPC scaffolds (Fig. 5a). Scanning electron microscopy (SEM) photos revealed that the transfected ADSCs were tightly adherent to the surface and pores of the CPCs with a large amount of ECM fibril networks (Fig. 5b).

We reconstructed the new bone by micro-CT at 8 weeks post-operation. New bone formation was elevated by implantation of the Lenti-circRNA-vgll3-transfected ADSCs composition with CPCs (Fig. 5c). Quantitative analysis revealed that bone mineral density (BMD) in the Lenti-NC skulls was 0.51 ± 0.01%, that in the Lenti-circRNA-vgll3 group (0.66 ± 0.04%) was markedly higher (*P* < 0.05), and that in the circRNA-vgll3 inhibitor group (0.36 ± 0.04%) was lower (*P* < 0.05) (Fig. 5d). The bone volume/tissue volume (BV/TV) and trabeculae number (Tb. N) analyses showed the similar pattern (Fig. 5e, f). These data suggest that circRNA-vgll3 positively regulates bone regeneration of ADSCs in vivo.

**Fig. 6:**
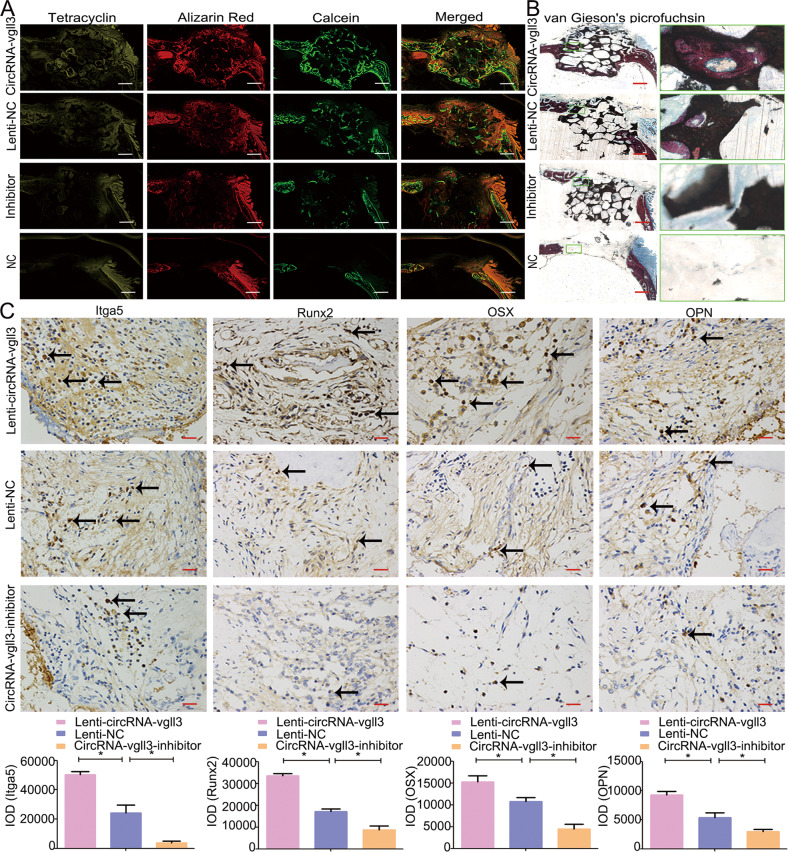
Fluorochrome-labeling and histological analysis of in vivo bone regeneration. **a** The area of Tetracycline-labeled, Alizarin Red-labeled and Calcein-labeled bone was elevated by Lenti-circRNA-vgll3 group, whereas the circRNA-vgll3 inhibitor reduced this area. Scale bars: 1000 µm. **b** van Gieson’s picrofuchsin staining showed that Lenti-circRNA-vgll3 increased the new bone percentage in the scaffold tissue whereas the circRNA-vgll3 inhibitor impaired it. Scale bars: 1000 µm. **c** Immunohistochemical analysis of Itga5, Runx2, OSX and OPN in the defect area at 8 weeks post-operation. Scale bars: 25 µm. *P < 0.05, statistical analysis was performed by one-way ANOVA.

Confocal laser scanning microscopy (CLSM) imaging was used to follow up the fluorochrome-labeled new bone area (Fig. 6a). The area of Tetracycline, Alizarin Red and Calcein-labeled bone was elevated in the Lenti-circRNA-vgll3 group. Van Gieson’s staining showed that Lenti-circRNA-vgll3 increased the new bone volume in the scaffold tissue compared to Lenti-NC (Fig. 6b). Besides, immunohistochemistry for Itga5, Runx2, OSX, and OPN in the surrounding bone tissue, which exhibited brown staining, was conducted, and assessed for integrated optical density (IOD) (Fig. 6c). The IOD for Itga5, Runx2, OSX, and OPN in the Lenti-circRNA-vgll3 group (50420 ± 2036.4, 33591.1 ± 1031.8, 15255.1 ± 1409.7, and 9253.6 ± 640.5, respectively) was higher than those in the Lenti-NC group (24245 ± 5491.7, 17076.1 ± 1252.3, 10747.7 ± 954.4, and 5354.7 ± 864.0, respectively), whereas the IOD in the circRNA-vgll3 inhibitor group (3901.3 ± 1231.0, 8735.3 ± 1818.4, 4481.5 ± 1133.0, and 2970.4 ± 373.6, respectively) was lower. Altogether, these data reveal that circRNA-vgll3-modified ADSCs markedly enhanced new bone formation in vivo, suggesting their application potential in treating non-healing bone defects.

**Fig. 7:**
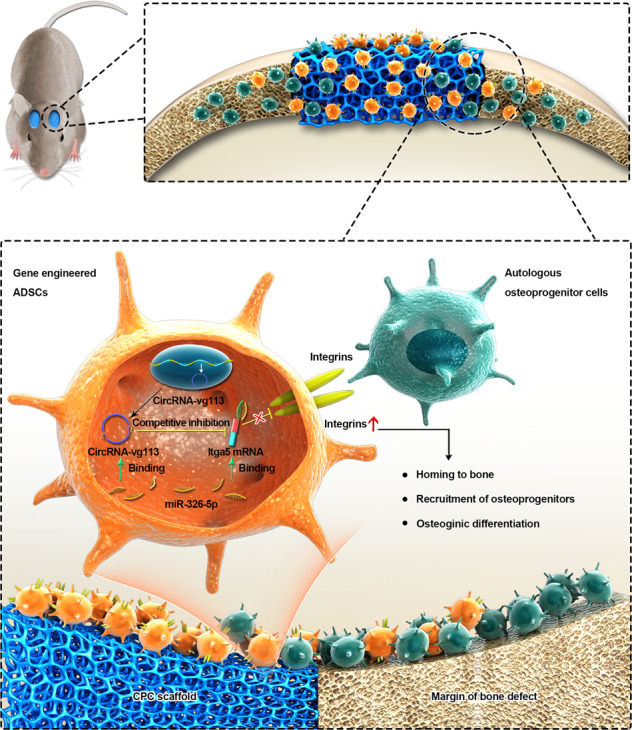
A model for circRNA-vgll3-modified ADSCs in bone regeneration. CircRNA-vgll3-modified ADSCs composition with CPC scaffolds promote the repair of critical-sized bone defects. CircRNA-vgll3 directly sequesters miR-326-5p in the cytoplasm and inhibits its activity, which in turn upregulates the protein levels of Itga5. The upregulated Itga5 protein levels help mediate the homing of ADSCs to the bone, the recruitment of osteoprogenitor cells, and the promotion of osteogenic differentiation in ADSCs.

We summarized a model for circRNA-vgll3-modified ADSCs composition with CPC scaffolds in repairing critical-sized bone defects (Fig. 7). CircRNA-vgll3 directly sequesters miR-326-5p in the cytoplasm and inhibits its activity, which in turn upregulates the protein levels of Itga5. The upregulated Itga5 levels help mediate the homing of ADSCs to the bone, the recruitment of osteoprogenitor cells, and the promotion of ADSC osteogenic differentiation.

## Discussion

Increasing evidences suggest that circRNAs are important and functional in regulating cellular behavior [8, 17]. However, no eviendence demonstrated the role of circRNAs in ADSC osteogenesis and the underlying mechanisms. In current study, our data exhibit that circRNA-vgll3 obviously promoted ADSC osteogenic differentiation by functioning as a miRNA sponge to attenuate the miRNA-326-5p-mediated inhibition of Itga5. CircRNA-vgll3 originates from the vgll3 locus. The vgll3 locus, which is related to the transcriptional cofactor Vestigial, is involved in cell fate determination in muscle development [40] and mesenchymal cell fate determination toward adipogenesis and osteogenesis [41]. The transcriptional regulation mechanism of vgll3 and circRNA-vgll3 need further validation.

As gene transfer strategies have become one of the highly effective tissue engineering approaches to support repair in many organ systems [6], the functional role of circRNA-vgll3 introduces the idea of using circRNA-vgll3-modified seed cells for bone repair. The most intensively studied gene transfer object is cDNA such as that of the osteogenic gene, BMP2 [42]. In addition to cDNAs, RNAs can also be delivered by transfer vectors and be used for regeneration purposes. Of these, miRNAs have gained attention because they are involved in the post-transcriptional regulation of gene expression, and many are correlated with bone metabolism [43]. Our team have revealed that miRNAs, including miR-31, miR-135, miR-26a, etc., can regulate osteogenic differentiation positively or negatively [26–29]. Compared to linear RNAs, circRNAs are more stable and longer lasting, because they lack a free end for RNA enzyme-mediated degradation [9].

The mechanisms for circRNAs involvement in osteogenesis remain unclear. The best-known function pattern for circRNAs is serving as miRNA ‘sponges’ [8]. It was reported that the relative abundance of circRNAs and miRNAs, the stability of circRNAs and the potential miRNA response elements (MREs) in circRNAs contributed to circRNAs and miRNAs ‘sponging’ crosstalk [44]. Considering the abundance, stability and the number of MREs of circRNA-vgll3, we speculate a ‘sponging’ model for circRNA-vgll3 and miR-326-5p to interact in ADSCs. This model has been validated in some stem cell research studies. For example, Yu et al. suggested that circBIRC6 “sponges” miR-34a and miR-145 to modulate embryonic stem cell pluripotency as well as differentiation [12]. CircZNF91 contains target sites for miR-23b-3p to regulate keratinocyte differentiation [13]. By ‘sponging’ miR-378a-3p, circLMO7 regulates myoblast differentiation and survival [45]. CircRNA-vgll3 regulates osteogenic differentiation in ADSCs by directly targeting miR-326-5p, which in turn blocks osteogenic differentiation by suppressing Itga5 translation. Along with miRNAs and their targets, the circRNA-miRNA-mRNA axis may serve as an extensive network to regulate gene expression, which requires further study to elucidate the underlying mechanisms.

Itga5 belongs to the integrin receptor family, which mediates cell attachment [36]. In this study, circRNA-vgll3 could significantly promote the expression of Itga5; thus, circRNA-vgll3-modified ADSCs may have an increased ability for cell attachment, including homing to the bone, and recruiting osteoprogenitor cells. Moreover, Itga5 is closely involved in the bone formation process [30]. A previous study exhibited that lentivirus-mediated expression of Itga5 in MSCs promoted the repair of cranial bone defects after implantation in mice [46]. In this study, circRNA-vgll3-modified ADSCs in combination with CPC scaffolds significantly promoted new bone formation. We deduced that this is resulted from the action of continuously upregulated Itga5 on the enhanced attachment to bone, the elevated osteoprogenitor cells recruitment and the promoted osteogenic differentiation.

The difficulties in repairing non-healing fractures motivates the development of gene modification techniques within tissue engineering. CircRNAs are cellular targets valuable for the exploitation of particular medical interventions [47], and this study indicates that circRNA-transduced ADSCs hold potential for repairing non-healing bone defects. However, circRNA-based therapeutics require an in-depth understanding of their multiple functions in gene regulation, which require further investigation in the future.

## Conclusion

In this study, our data demonstrate a new regulatory role of circRNA-vgll3 in the osteogenic differentiation of ADSCs through a circRNA-vgll3/miR-326-5p/Itga5 pathway, and that circRNA-vgll3-modified ADSCs can markedly enhance new bone formation in vivo. This study provides new insights underlying the effects and regulatory mechanisms of circRNAs in ADSC osteogenesis, and moreover, provides preclinical data supporting the potential application of circRNA-vgll3-engineered ADSCs in bone regenerative medicine.

## Methods and materials

### ADSC isolation and identification

The Shanghai Animal Experimental Center provided Sprague Dawley rats. In this study, we abided by all the regulations of the Animal Research Committee of Ninth People’s Hospital, Shanghai Jiao Tong University School of Medicine. Isolation of ADSCs was based on a previously reported protocol [26]. Osteogenic differentiation of ADSCs was induced using BMP2 (200 ng/ml, R&D, Minneapolis, MN, USA), with circRNA-vgll3 or miR-326-5p lenti-virus transfected or not. ADSCs were characterized by flow cytometry with CD29, CD90, CD45, and CD31 (All from BD Biosciences, San Jose, CA, USA) staining.

### RNA-seq analysis

RNA-seq libraries were constructed as follows: extracted total RNA was exposed to a Ribo–Zero kit to digest the ribosomal RNA; interrupt reagent was added to fragment the RNA into short pieces. The short RNA pieces were used as templates and random hexamers were used as primers to synthesize the first cDNA chain. To remove the second strand, a second strand synthesis reaction system was prepared. The resulting first-strand cDNA underwent PCR amplification of 13–16 cycles. We sequenced the libraries on an Illumina sequencing platform (HiSeqTM 2500 platform), to generate paired-ended 150 bp/125 bp reads. The resulting data were uploaded in SRA (SRP165832).

### Lentiviral construction and transduction

Rat circRNA-vgll3 genes are located on chromosome 11 p31.4. The oligonucleotide sequence was synthesized as indicated in Table 1 and cloned into the pHBLV-CMV-Cicr-MCS-EF1-ZsGreen-T2A-puro plasmid (Hanheng Biology, Shanghai, China). The integration of transfected virus in genomics was demonstrated by detection of ZsGreen and puro tag sequence of the lenti-virus in the genomics using the NGS technology. The inhibition fragment used synthetic shRNA sequences (shown in Table 1) and was cloned into the pHBAd-U6-MCS-CMV-GFP vector. The pSPAX2, pMD2G and target gene plasmids were transfected together into 293Ts with the supernatants collected, filtered, and concentrated after 48 h. For lentiviral transfection, Opti-MEM (Invitrogen Carlsbad, CA, USA) was used as the culture medium with a suitable volume of viral supernatant and 5 mg/ml polybrene (Hanheng Biology) added.

**Table 1:** Constructed sequences used in this study.

Name	Sequence (5′-3′) Forward (5-3′) Annealing temperature (°C) Product size (base pairs)
CircRNA-vgll3	ACAGCTCAGCTCTTTCAAGCCAGCGGAGTAATTTTCCAACTTCCTTTTGGACCAGCTCTTACCAGCCCCCACCGGCACCTTGTTTGGGGGGAGTTCATCCTGACTTCCAAGTCACTGCACCCCACGGCACCTTTACAACAGCAGACCCCAACTCTTGGCCAGGACATGGTCTGCATCAGACTGGCCCTGCCCCACCCCCTACTGCATCTGAGTCTTGGCACTATCCTCTGGCATCTCAGGTGAGCCCATCCTACAGCCACATGCATGACATGTACATGCGGCATCACCATCCTCATGCTCACATGCACCACCGCCACCACCACCATCACCACCACCCAACTGCTGGCTCTGCCCTGGATCCCGCGTATGGGCACCTGCTGATGCCATCAGTGCGTGCTGCCAGGATTCCTGCTCCCCAGTGTGACATCACAAAGACAGATCTGACTACAGCCACCACTGCTACCTCAGCATGGGCCGGAGCCTTTCATGGGACAGTGGACATCGTGCCAAGTGTGGGCTTCGAAACAG
CircRNA-vgll3-shRNA1	GGCTTCGAAACAGACAGCTCAGCTC
CircRNA-vgll3-shRNA2	GTGGGCTTCGAAACAGACAGCTCAG
CircRNA-vgll3-shRNA3	CGAAACAGACAGCTCAGCTCTTTCA
Si-vgll3	Sense: CCCUACAGCCUGCUAUCAUTT antisense: AUGAUAGCAGGCUGUAGGGTT
miR-326-5p	CAGGCCCTGGACTCAGCTGAGCTTGACTACTTCAGAAGCTGTGGCAAGACACGCAGACCTGGACCAGGGAGGGCTCTGCTCCAGAAGAGTTCTTCCGAGTCTCATCTGTCTGTGGGGCTGGGGGCAGGGCCTTTGTGAAGGCGGGTTATGCTCAGATCGCCTCTGGGCCCTTCCTCCAGTCCCGAGGCAGATTTACCTCGAGGCAAAGCAGCACACCAGCTCCAGGCTCCCTCAGGGCCTCATCTTTTGTGTCCCAGGTCTCAGAGCTCGCCTGAATTTGTAAAAGAAAGATCGTTTTTT
miR-326-5p inhibitor	TTCACAAAGGCCCTGCCCCC
Itga5	CCTGGAACCTCAGCCTGACCAAACAGGCTCACTCATTCCCCAGAGAACGGGAAGCCCCAGGCCTGTACGACCTGGGTTCTGCCCACCAGCTGCACTGACGCTGCCCCTCTCTCCCTAGCCAACCCTCCCCTCTCCTCCAACCCCCCCAACTTATTTAAACTCTGTTGCAAGTGCAATAAACCCCACTCACTGCCCCACTGACC
Itga5 mut	CCTGGAACCTCAGCCTGACCAAACAGGCTCACTCATTCCCCAGAGAACGGGAAGCCCCAGGCCTGTACGACCTGGGTTCTGCCCACCAGCTGCACTGACGGACGGGGTCTCTCCCTAGCCAACCCTCCCCTCTCCTCCAACCCCCCCAACTTATTTAAACTCTGTTGCAAGTGCAATAAACCCCACTCAGACGGGGCTGACC
circRNA-vgll3-wt 1	TTTGGGGGGAGTTCATCCTGACTTCCAAGTCACTGCACCCCACGGCACCTTTACAACAGCAGACCCCAACTCTTGGCCAGGACATGGTCTGCATCAGACTGGCCCTGCCCCACCCCCTACTGCATCTGAGTCTTGGCACTATCCTCTGGCATCTCAGGTGAGCCCATCCTACAGCCACATGCATGACATGTACATGCGGCATCACCATCCTCATGCTCACATGCACCACCGCCACCACCACCATCACCACCACCCAACTGCTGGCTCTGCCCTGGATCCCGCGTATGG
circRNA-vgll3-mut 1	TTTGGGGGGAGTTCATCCTGACTTCCAAGTCACTGCACCCCACGGCACCTTTACAACAGCAGACCCCAACTCTTGGCCAGGACATGGTCTGCATCAGACTCCGGGACGGGGACCCCCTACTGCATCTGAGTCTTGGCACTATCCTCTGGCATCTCAGGTGAGCCCATCCTACAGCCACATGCATGACATGTACATGCGGCATCACCATCCTCATGCTCACATGCACCACCGCCACCACCACCATCACCACCACCCAACTGCTCCGAGACGGGAGGATCCCGCGTATGG
circRNA-vgll3-wt 2	CCACCACCACCATCACCACCACCCAACTGCTGGCTCTGCCCTGGATCCCGCGTATGGGC
circRNA-vgll3-mut 2	CCACCACCACCATCACCACCACCCAACTGCTCCGAGACGGGAGGATCCCGCGTATGGGC
circRNA-vgll3-wt	AGGACATGGTCTGCATCAGACTGGCCCTGCCCCACCAACTGCTGGCTCTGCCCTGGATCCCGCGTATGGGC
circRNA-vgll3-mut	AGGACATGGTCTGCATCAGACTCCGGGACGGGGACCAACTGCTCCGAGACGGGAGGATCCCGCGTATGGG
Itga5-shRNA1	TGGCTATGTCACTGTCCTTAA
Itga5-shRNA2	TGGCATGCGCTCCACTGTATA
Itga5-shRNA3	GGGCACCCAAGGCTAACACTA
Itga5-shRNA4	GCAGATCTCGGAGTCCTATTA

Lentiviral construction and transduction of miR-326-5p was similar to the above protocols. The lentiviral expressing miR-326-5p was Lenti-miR-326-5p. We directly synthesized miR-326-5p, which is listed in Table 1 (in bold and underlined for the stem loop region) and cloned the sequences into the GV309 plasmid (Genechem Technology, China). The reverse complementary sequence (as shown in Table 1) was cloned into plasmid (GV280, Genechem) to construct the Lenti-miR-326-5p inhibitor. Lenti-miR-NC was the empty lentiviral system with no insertion.

### RNA preparation and qPCR

According to the manufacturer’s description, Rneasy Midi kit (Qiagen, Redwood City, CA. USA) was used for ADSC nuclear and cytoplasmic RNA extraction. We used Trizol (Life, Carlsbad, CA, USA) reagent for isolating total RNA at osteogenic day 7 or specially indicated days. For the Rnase R digestion experiment, we treated RNA with RNase R (3 U/µg, Epicentre, Madison, WI, USA) for 20 min. For the actinomycin D (APExBIO, Houston, TX, USA) experiment, 1 μM actinomycin D was added to ADSCs and RNA was isolated at 0 h, 6 h, 12 h, 18 h and 24 h, respectively. To detect mature miRNA, a stem loop reverse transcription kit (BioTNT, Shanghai, China) was used, and specific primers for the stem loop reverse transcription are shown in Table 2. qPCR for detecting circRNA and mRNA expression levels was performed by SYBR Green PCR Mix (Applied Biosystems, Forstercity, CA, USA) and a QuantStudio TM 6Flex Real-Time PCR system (Applied Biosystems). BioTNT MicroRNA assay (BioTNT) was used to detect the level of mature miRNAs. After normalization to GAPDH (for mRNA and circRNA) or RNU6B (for miRNA), respectively, the RNA expression was presented in the form of relative fold change to the controls. For the actinomycin D experiment, the relative expression fold changes of circRNA-vgll3 or vgll3 at indicated time points (6 h, 12 h, 18 h, 24 h) were compared to the expression of circRNA-vgll3 or vgll3 at time point of 0 h, respectively. For the nucleoplasmic separation experiments, GAPDH was used as internal reference for cytoplasmic fraction, and RNU6B was used as internal reference for nuclear fraction. All primers are listed in Table 2 (mRNAs and circRNAs) and Table 3 (miRNAs).

**Table 2:** Primers used for quantitative RT-PCR.

Genes	Forward (5′-3′)	Reverse (5-3′)	Annealing temperature (°C)	Product size (base pairs)
Col1a1	GACTGTCCCAACCCCCAAAA	CTTGGGTCCCTCGACTCCTA	60	102
Runx2	ATCATTCAGTGACACCACCA	GTAGGGGCTAAAGGCAAA AG	60	141
OSX	GGA AAA GGAGGCACA AAGAA	CAGGGG AGA GGAGTCCAT T	60	106
OPN	CCAGCCAAGGACCAACTACA	AGTGTTTGCTGTAATGCGCC	60	132
OCN	CAGACA AGTCCCACACAGCA	CCAGCAGAGTGAGCAGAG AG	60	76
Itga5	CAG ATAGCA CCCGAGTTACCA	ACAATCCTACCTGCCCTA ACG	60	113
Vgll3	CCAACACCTTGCATCCCGAA	GGTCTGCTGTTGTAAAGGTGC	60	213
AdipoQ	CCACCCAAGGAAACTTGTGC	GACCAAGAACACCTGCGTCT	60	136
SOX9	GTCGGTGAAGAATGGGCAAG	GACCCTGAGATTGCCCGGA	60	161
GAPDH	AAGAAACCCTGGACCACCCAGC	TGGTATTCGAGAGAAGGGAGGG	60	178
CircRNA-vgll3	GGAGCCTTTCATGGGACAGT	CTGCTGTTGTAAAGGTGCCGTG	60	196

**Table 3:** Primers used for miRNA Stem loop reverse transcription and quantitative RT-PCR.

Genes	Stem loop reverse transcription	Forward (5′-3′)	Reverse(5-3′)
rno-miR-326-5p	CTCAACTGGTGTCGTGGAGTCGGCAATTCAGTTGAGTTCACAAA	CAGGGCCTTTGTGAA	TCAACTGGTGTCGTGG
rat Rnu6B	CTCAACTGGTGTCGTGGAGTCGGCAATTCAGTTGAGAAAATATG	CAAATTCGTGAAGCGTT	TGGTGTCGTGGAGTCG

### Western blot analysis

The RIPI (Life) and protease inhibitor cocktail (Roche; Basel, Switzerland) were used for protein cracking at osteogenic day 7 or specially indicated days. Electrophoresis, transferring and blocking were performed as previously. Primary antibodies used include antibodies against Itga5 (Santa Cruz, Dallas, TX, USA, SC-166665), Runx2 (Abcam, Cambridge, MA, USA, ab23981), OPN (Abcam, ab8448), BSP (Abgent, San Diego, CA, USA, ap14114a), OSX (Abcam, ab22552), OCN (Abcam, ab13418), COl1a1 (Abcam, ab34710), CRY2 (Proteintech, Rosemont, IL, USA, 13997-1-AP), Mapk3 (Cell Signaling Technology Inc, Boston, Mass, USA, 9102) and internal reference β-actin (Proteintech, Rosemont, IL, USA, 66009-1). Afterwards, they were incubated with respective secondary antibodies (Sigma-Aldrich, Saint Louis, MO, USA). The bands were viewed using an Odyssey image scan system (Nikon, Tokyo, Japan) or a Tanon image viewer system (Shanghai, China).

### RNA pull-down assay

We obtained the biotin-labeled circRNA-vgll3 probe (5′-TGCCAAGTGTGGGCTTCGAAACAGACAGCTCAGCTCTTTCAAGCCAGCGGAGTAA-3′-biotin) and mock probe (5′-TTACTCCGCTGGCTTGAAAGAGCTGAGCTGTCTGTTTCGAAGCCCACACTTGGCA-3′-biotin) from Sangon Biotech, and performed the RNA pull-down assay according to a previous report [48]. 1% formaldehyde was used for fixation of cells. After lysis, sonication and centrifugation, 50 μl supernatant was pipetted for input, with the remaining incubated with biotin-labeled probe and streptavidin dynabeads (M-280, Invitrogen) overnight. Proteinase K was used to reverse the crosslinking on the next day. Finally, RNA extraction was performed using TRIzol for later detection.

### RIP experiment

Abiding by the Magna RIP Kit (Millipore, Billerica, MA, USA) instruction, RIP lysis buffer plus RNase inhibitors and protease inhibitors were used for lysis. The ADSCs were lysed by 250 μl lysis buffer. We pipetted 20 μl cell lysis for the input for western blotting, and another 20 μl cell lysis for the input for qPCR. Then we pipetted 100 μl cell lysis to experience immunoprecipitation by anti-Ago2-magnetic bead or anti-IgG-magnetic bead. After over-night immunoprecipitation, 10% of the anti-Ago2-magnetic bead suspension or anti-IgG-magnetic bead suspension was pipetted as anti-Ago2 or anti-IgG group for western blotting. Meanwhile, 90% of the anti-Ago2-magnetic bead suspension or anti-IgG-magnetic bead suspension was pipetted as anti-Ago2 or anti-IgG group for qPCR. The immunoprecipitated RNAs were extracted and reverse transcribed after proteinase K treatment for the detection of miRNAs and circRNAs.

Plasmids containing circRNA-vgll3 and miR-326-5p fragments were synthetized to serve as circRNA-vgll3 and miR-326-5p standards (produced by Genephama, China). According to the known mass concentration (ng/μl), base number (bp) of the standards and average molecular mass of bases, we can convert the mass concentration of standards to molecular copy numbers per μl (copies/μl). The circRNA-vgll3 and miR-326-5p standards were then experienced 10 times gradient dilution. Next, the gradient diluted standards and the immunoprecipitated samples were experienced qPCR. Based on the measured CT values (Cycle threshold values) of standards and known concentration of standards, we can build a standard curve for the log (copy number) and CT values. Based on the CT values of immunoprecipitated samples, we can calculate the circRNA-vgll3 or miR-326-5p copy numbers in immunoprecipitated samples.

### Luciferase assay

The predicted target sequences of miR-326-5p in Itga5 or their corresponding mutation were cloned behind the luciferase reporter sequences. The predicted targets of miR-326-5p in circRNA-vgll3 and their corresponding mutation were designed as follows: in the downstream of the luciferase coding region, the 211 bp fragments in circRNA-vgll3 containing the binding site 1 for miR-326-5p were inserted, which was called circRNA-vgll3-wt 1 and the lucefarase plasmid containing the corresponding mutation sequence was called circRNA-vgll3-mut 1; in addition, in the downstream of the luciferase coding region, the 59 bp fragments in circRNA-vgll3 containing the binding site 2 for miR-326-5p were inserted, which was called circRNA-vgll3-wt 2 and the lucefarase plasmid containing the corresponding mutation sequence was called circRNA-vgll3-mut 2; meanwhile, in the downstream of the luciferase coding region, the fragments containing the binding sites 1 and 2 for miR-326-5p were inserted, which was called circRNA-vgll3-wt and the lucefarase plasmid containing the corresponding mutation sequence was called circRNA-vgll3-mut.

The luciferase reporter plasmid, miR-326-5p plasmid were then co-transfected into 293T cells or ADSCs. Luciferase Reporter Assay (Promega, Madison, WI, USA) was performed to detect the Luciferase activities at 48 h post-transfection.

### FISH

Bone tissue was fixed for 24 h in RNAase free formalin and decalcified in RNAase free Ethylene Diamine Tetraacetic Acid solution for 1month after separating from the 1 month rats. The separated adipose tissue was fixed in RNAase free formalin for 24 h and experienced cryo-section. ADSCs were fixed in 4% RNAase free paraformaldehyde (PFA) for 15 min. After fully hydration, 1 part of 30% H_2_O_2_ + 9 parts of pure methanol mixture was added for 10 min. 0.2 mol/L hydrochloric acid was dropped onto the slide for 15 min at room temperature. Add Proteinase K and incubate in molecular hybridization meter at 37 °C for 20 min. Then cells were incubated with 500 ng/ml FAM-labeled and TYE-labeled probe in hybridization buffer at 37 °C overnight. MiR-326-5p probe was obtained from EXIQON YD00614165-BCO. The sequence of FAM-labeled circRNA-vgll3 probe was: 5′-FAM- -CUGUUUCGAAGCCCACACUUGGCACGAUGUCCACUGUCCCAUGAA - 3′.

### ALP and ARS staining

ALP and ARS staining was performed at osteogenic day 7 and 14 respectively, as previously reported [26]. Semiquantitative analyses of ALP were performed using an Alkaline Phosphatase Assay Kit (Beyotime). The activity was measured using a spectrophotometer (ELX800, BioTek, USA) at a wavelength of 405 nm. Semiquantitative analysis of ARS was assessed by adding 10% cetylpyridinium chloride (Sigma-Aldrich) and reacting at 37 °C for 30 min before absorbance reading at 590 nm.

### CLSM and SEM imaging

To detect immunofluorescence, after fixation, permeabilization and blocking, the ADSCs at osteogenesis day 7 were incubated overnight at 4 °C with antibodies including anti-OPN (1:200, Abcam), anti-BSP (1:200, Abcam), anti-Runx2 (1:200, Abcam), and anti-Itga5 (1:50, Santa Cruz). They were incubated with a secondary antibody (Alexa Fluor 546, 1:800, Invitrogen) the next day. Hochest (Invitrogen) was used for Cell Nucleus staining before viewing on a CLSM Nikon A1 microscope (Nikon). SEM was performed as reported earlier [49]. In short, 2.5% glutaraldehyde and graded ethanol solutions were used for fixation and dehydration. Samples were then air-dried, gold sputtered, and imaged on a SEM (Tescan VEGA 3 LMU, Tescan, Brno - Czech Republic).

### Animal experiments

CPC scaffolds were obtained from Rebone Inc. in Shanghai, China. ADSCs seeded onto the CPC scaffolds were transfected with Lenti-circRNA-vgll3 or circRNA-vgll3 inhibitor. As a previous developed method, after anesthetization, we created two 5 mm critical-sized defects on the exposed calvarium of 8-week old male Sprague Dawley rats, using an electric trephine (Nouvag AG, Goldach, Switzerland). The constructed CPC scaffolds were then implanted in the created model randomly and blindly.

### Sequential fluorescent labeling

The rats were intraperitoneally injected with 5 mg/kg Tetracycline (Sigma-Aldrich) 2 weeks post-operation. At 4 weeks, the rats were intraperitoneally injected with 0.8 ml/kg Alizarin Red (Sigma-Aldrich) (3% (w/v) in 2% (w/v) sodium bicarbonate solution) and at 6 weeks, 5 ml/kg Calcein (Sigma-Aldrich) (1% (w/v) in 2% (w/v) sodium bicarbonate solution) was intraperitoneally injected. The rats were sacrificed at 8 weeks for detecting the polychrome fluorescence.

### Micro-CT

The skulls of the sacrificed rats were fixed in 4% PFA. We reconstructed the skulls using micro-CT (Skyscan 1076, Bruker micro-CT, Kontich, Belgium) at 70 KV, 114 A, and an isotropic pixel size of 18 μm.

### Histological observation

After dehydration, the samples were embedded, cut, polished and observed under a CLSM (Nikon). The excitation/emission wavelengths of Tetracycline are 405/580 nm, those of Alizarin Red are 543/580-670 nm and those of Calcein are 488/500-550 nm. We then stained the sections with van Gieson’s picrofuchsin.

The remaining samples were decalcified for 2 weeks using 10% ethylene diamine tetraacetic acid. Immunohistochemistry for Itga5 (Santa Cruz); Runx2 (Abcam), OPN (Abcam) and OSX (Abcam) was then conducted and analyzed using Image Pro Plus.

### Statistical analysis

All experiments were conducted three times unless otherwise specified in this study. For data analysis, the obviously deviated data (outlier) was excluded based on Q test. The data are presented as mean ± standard deviations and the error bars represent the standard deviations. Statistical significance was calculated using one-way ANOVA or two-tailed Student’s *t* test. *P < 0.05 was considered statistically significant.

## Supplementary information

supplementary file

Supplementary Fig.1

Supplementary Fig.2

Supplementary Fig.3

Supplementary Fig.4

Supplementary Fig.5

Supplementary Fig.6

Supplementary Fig.7

Supplementary Fig.8

Supplementary Figure 9

Supplementary Figure 10

Supplementary Figure 11

Supplementary Figure 12

Supplementary Figure 13
