# Supplementary material for: CircRNA-vgll3 promotes osteogenic differentiation of adipose-derived mesenchymal stem cells via modulating miRNA-dependent integrin α5 expression
Source: Cell Death Differ. 2020 Aug 19;28(1):283–302. doi: 10.1038/s41418-020-0600-6 (PMC7853044; doi:10.1038/s41418-020-0600-6)
Supplement: Supplementary file 1 — supplementary file [file 41418_2020_600_MOESM1_ESM.doc]

**CircRNA-vgll3 promotes osteogenic differentiation of adipose-derived mesenchymal stem cells via modulating miRNA-dependent integrin α5 expression**

**Running title: CircRNA-vgll3-modified ADSCs for bone regeneration**

Dandan Zhang†, Ni Ni†, Yuyao Wang†, Zhimin Tang, Huiqin Gao, Yahan Ju, Na Sun, Xiaoyu He, Ping Gu*, and Xianqun Fan*

Department of Ophthalmology, Ninth People's Hospital, Shanghai Jiaotong University School of Medicine, Shanghai Key Laboratory of Orbital Diseases and Ocular Oncology, Shanghai, 200011, P.R. China

† These authors contributed equally to this work.

* To whom correspondence should be addressed:

Tel: +86 021 2327 1699*5587 Fax: +86 021 6313 7148

mail: fanxq@sjtu.edu.cn, guping2009@126.com,

Conflict of interest

There is no conflict of interest in this study.

**Supplementary Data**

**Supplementary Fig.1**

ADSCs were identified by Flow cytometry, showing a high expression of CD29 and CD90 and a low expression of CD45 and CD31.

**Supplementary Fig.2**

Heatmap of abundant exonic circRNAs in BMP2-induced ADSCs and naive ADSCs.

**Supplementary Fig.3**

A diagrammatic drawing for clarifying the position of inhibitors.

**Supplementary Fig.4**

The MOI selection for ADSC showed in MOI=20, 50 and 100 groups, the positive ratio of ZsGreen+ cells reached more than 80%. Scale bars: 100 µm.

**Supplementary Fig.5**

**(A).** The provided virus sequence was used as the reference genome sequence (ZsGreen: 696 bp; Puro: 600bp). The original disembarkation data were compared with the reference genome sequence. No virus sequence was found in the untreated ADSC samples. The transfected ADSCs contained a large number of ZsGreen and puro virus sequences, indicating that the sample's DNA contained viruses. **(B).** After the combination of ZsGreen and puro virus sequences, the Fa files were used as references. The data of detected rat sample sequences were compared to the combined reference genome using BWA. The results of the untreated ADSC sample showed no viral sequences integrated into the ADSC genome, while the transfected ADSCs were found to have an integration site (chrX: 77262913).

**Supplementary Fig.6**

qPCR analysis showed that the circRNA-vgll3-inhibitors did not affect the expression of the host gene vgll3.

**Supplementary Fig.7**

The potent binding sites of miR-326-5p on circRNA-vgll3.

**Supplementary Fig.8**

KEGG pathway enrichment in BMP2-induced ADSCs and naive ADSCs.

**Supplementary Fig.9**

Luciferase reporter assay showed that co-transfection of miR-326-5p with the constructed Itga5 3'UTR-wt plasmid obviously decreased luciferase activity in ADSCs.

**Supplementary Fig.10**

Luciferase reporter assay showed that co-transfection of the miR-326-5p plasmid with circRNA-vgll3-wt 1 plasmid significantly impaired the luciferase activity in 293Ts.

**Supplementary Fig.11**

Luciferase reporter assay showed that co-transfection of the miR-326-5p plasmid with circRNA-vgll3-wt 2 plasmid significantly impaired the luciferase activity in 293Ts.

**Supplementary Fig.12**

**(A).** The amplification plots for detection of circRNA-vgll3 stoichiometries using the gradiente diluted circRNA-vgll3 standards and Ago-2 immunoprecipitated samples. The black arrow indicates the amplification plots of Ago-2 immunoprecipitated samples. **(B).** The relationship between log (circRNA-vgll3 copy number) and CT values. The red point indicate the location of Ago-2 immunoprecipitated samples. **(C).** The amplification plots for detection of miR-326-5p stoichiometries using the gradiente diluted miR-326-5p standards and Ago-2 immunoprecipitated samples. The black arrow indicates the amplification plots of Ago-2 immunoprecipitated samples. **(D).** The relationship between log (miR-326-5p copy number) and CT values. The red point indicate the location of Ago-2 immunoprecipitated samples.

**Supplementary Fig.13**

qRCR results exhibited that in the osteogenic differentiation process of ADSCs, the endogenous levels of osteogenic makers Runx2, OPN were gradually upregulated, accompanied by an up-regulated expression of circRNA-vgll3, and a correspondingly gradually down-regulated expression of miR-326-5p.
