# Supplementary figures and images for: CircRNA-vgll3 promotes osteogenic differentiation of adipose-derived mesenchymal stem cells via modulating miRNA-dependent integrin α5 expression
Source: Cell Death Differ. 2020 Aug 19;28(1):283–302. doi: 10.1038/s41418-020-0600-6 (PMC7853044; doi:10.1038/s41418-020-0600-6)

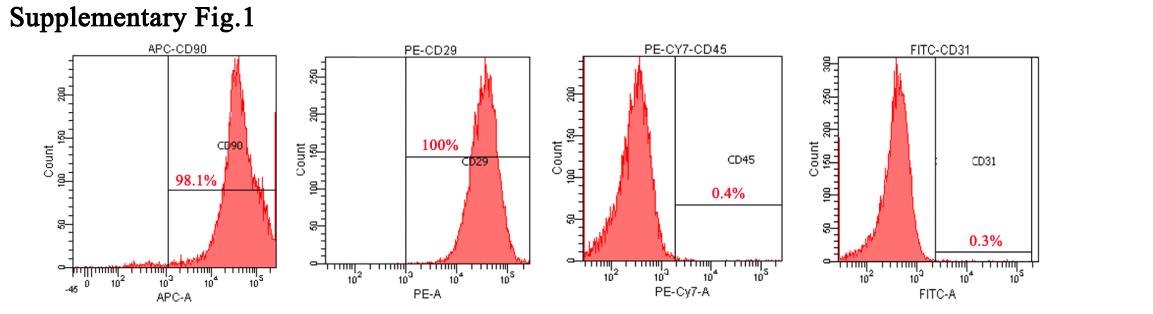

Supplement: Supplementary file 2 — Supplementary Fig.1 [file 41418_2020_600_MOESM2_ESM.tif]

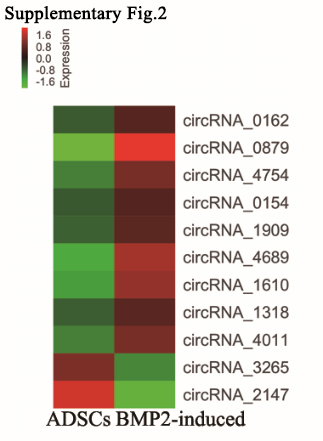

Supplement: Supplementary file 3 — Supplementary Fig.2 [file 41418_2020_600_MOESM3_ESM.tif]

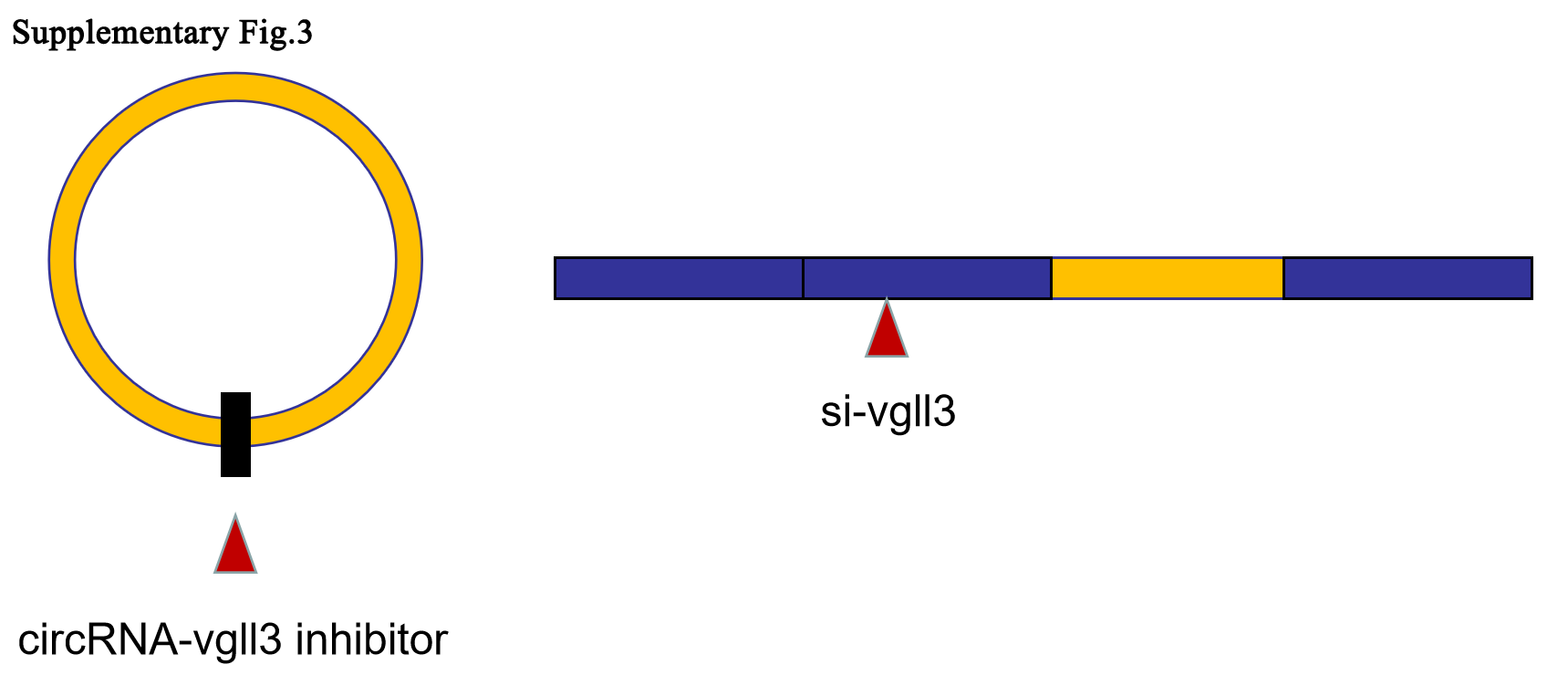

Supplement: Supplementary file 4 — Supplementary Fig.3 [file 41418_2020_600_MOESM4_ESM.tif]

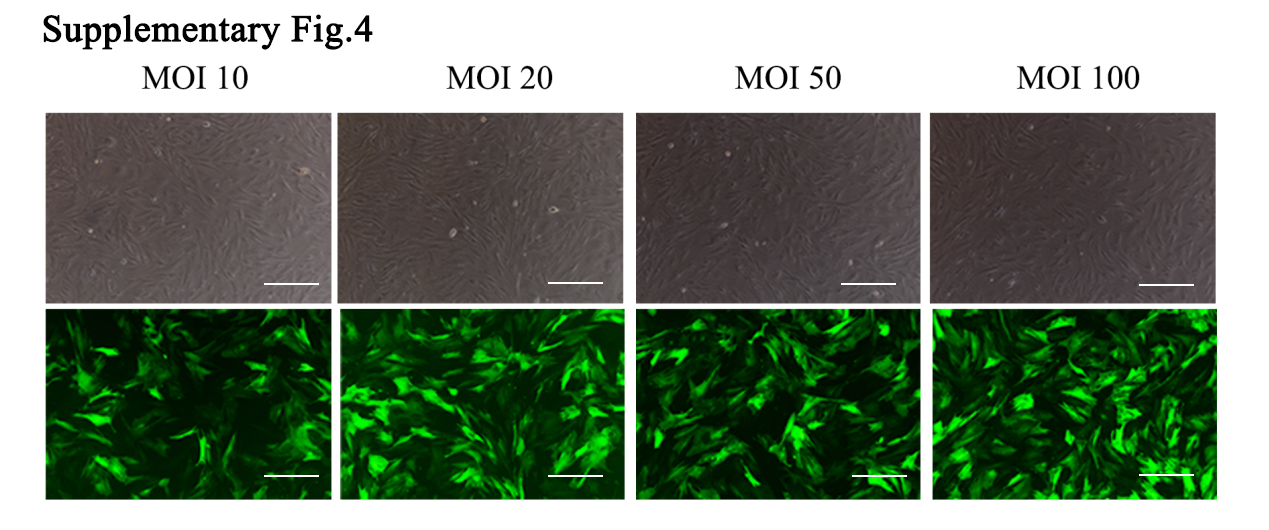

Supplement: Supplementary file 5 — Supplementary Fig.4 [file 41418_2020_600_MOESM5_ESM.tif]

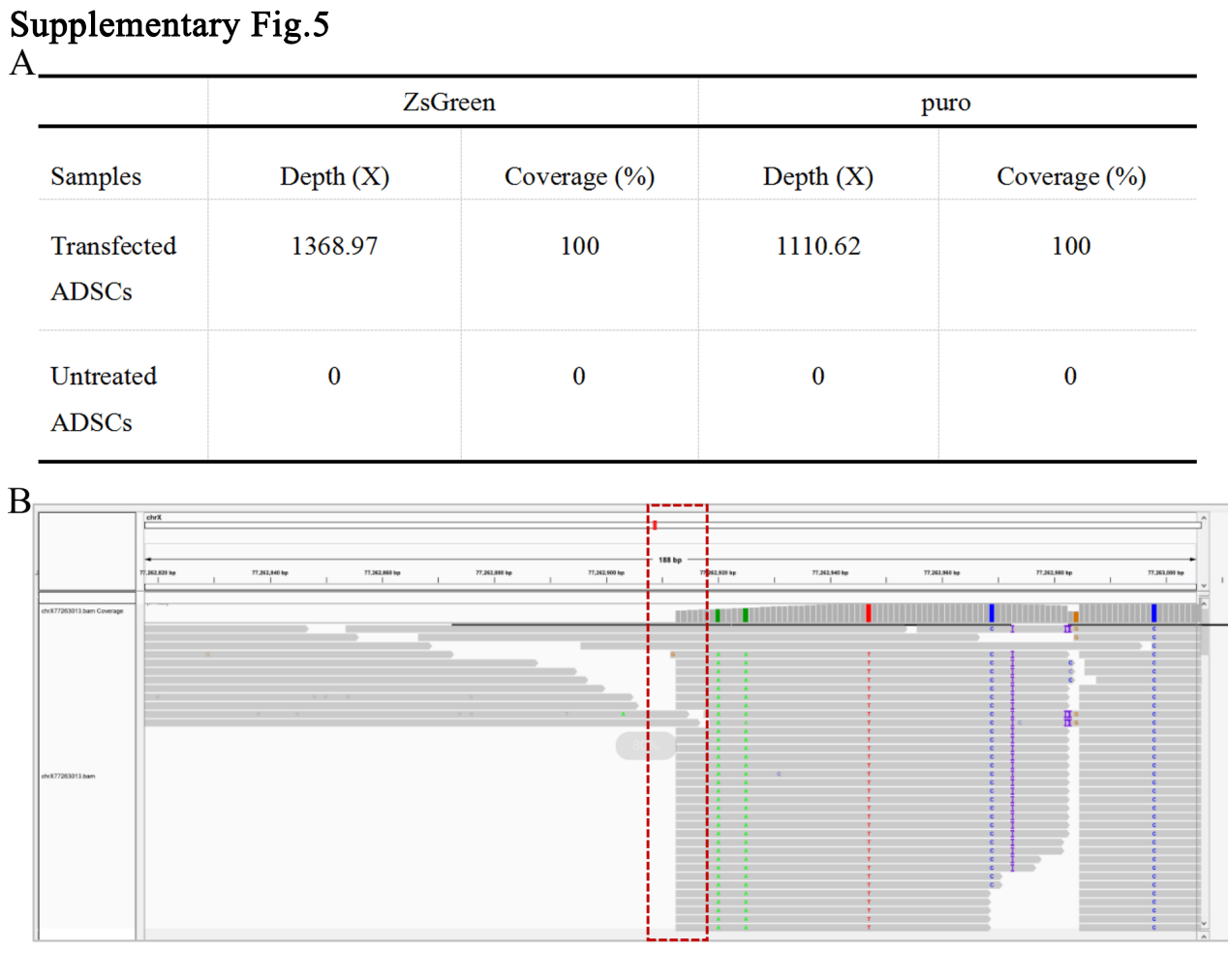

Supplement: Supplementary file 6 — Supplementary Fig.5 [file 41418_2020_600_MOESM6_ESM.tif]

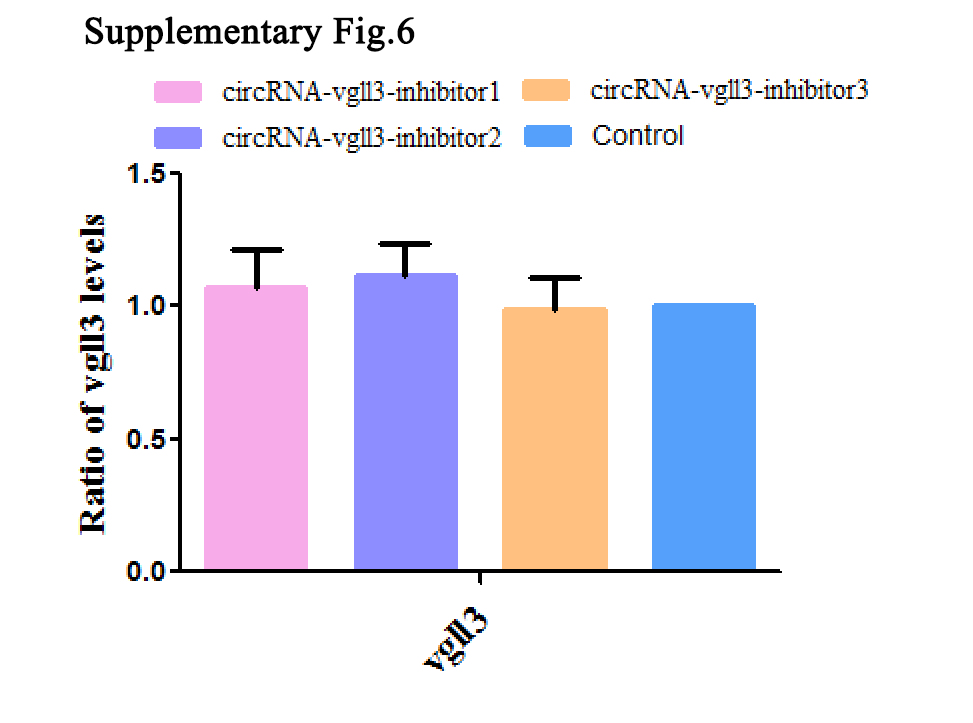

Supplement: Supplementary file 7 — Supplementary Fig.6 [file 41418_2020_600_MOESM7_ESM.tif]

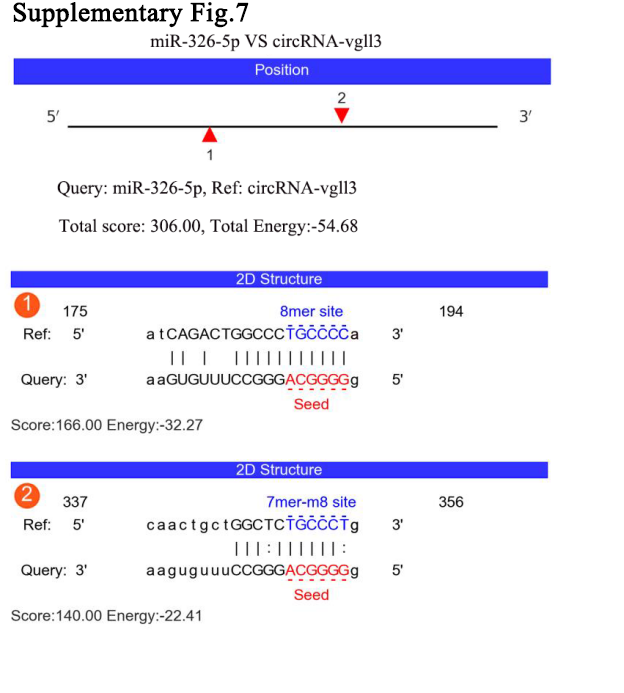

Supplement: Supplementary file 8 — Supplementary Fig.7 [file 41418_2020_600_MOESM8_ESM.tif]

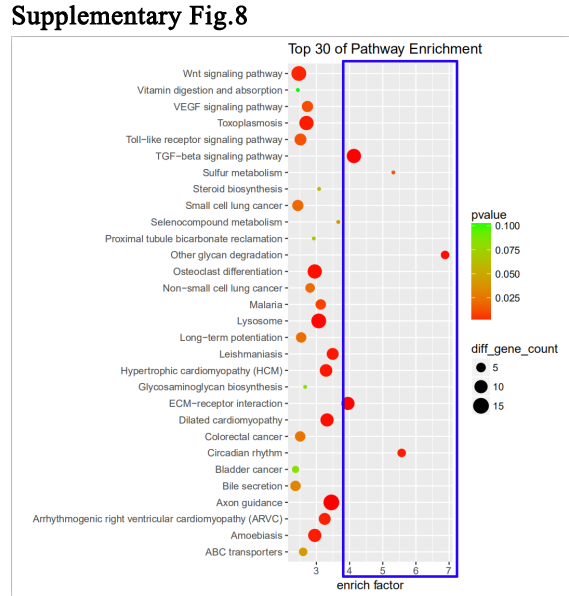

Supplement: Supplementary file 9 — Supplementary Fig.8 [file 41418_2020_600_MOESM9_ESM.tif]

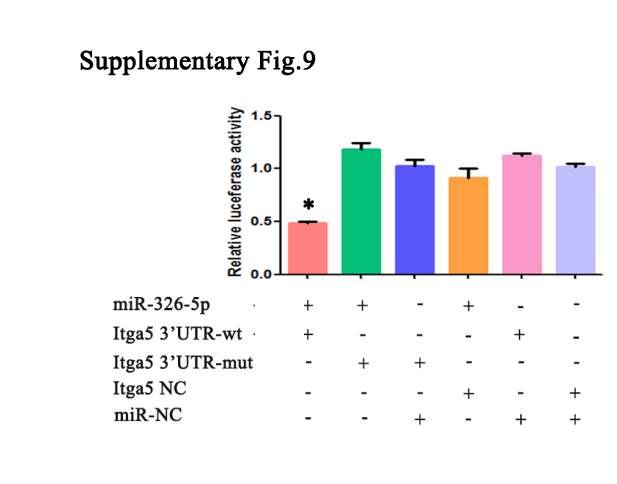

Supplement: Supplementary file 10 — Supplementary Figure 9 [file 41418_2020_600_MOESM10_ESM.tif]

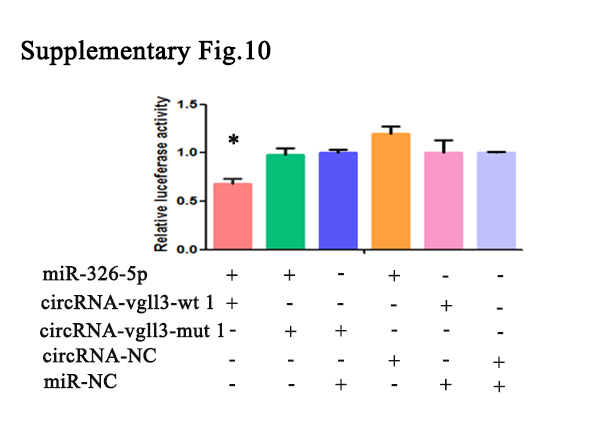

Supplement: Supplementary file 11 — Supplementary Figure 10 [file 41418_2020_600_MOESM11_ESM.tif]

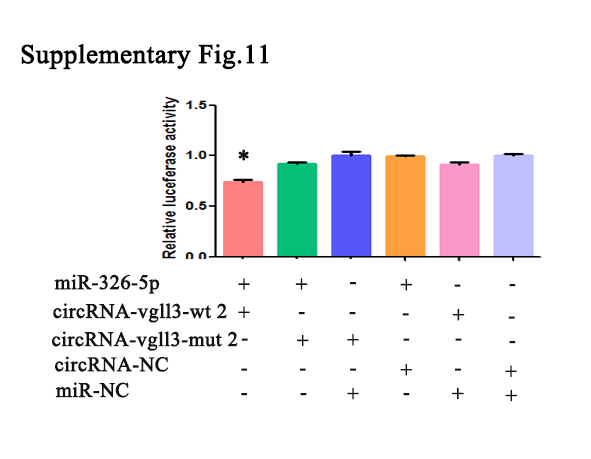

Supplement: Supplementary file 12 — Supplementary Figure 11 [file 41418_2020_600_MOESM12_ESM.tif]

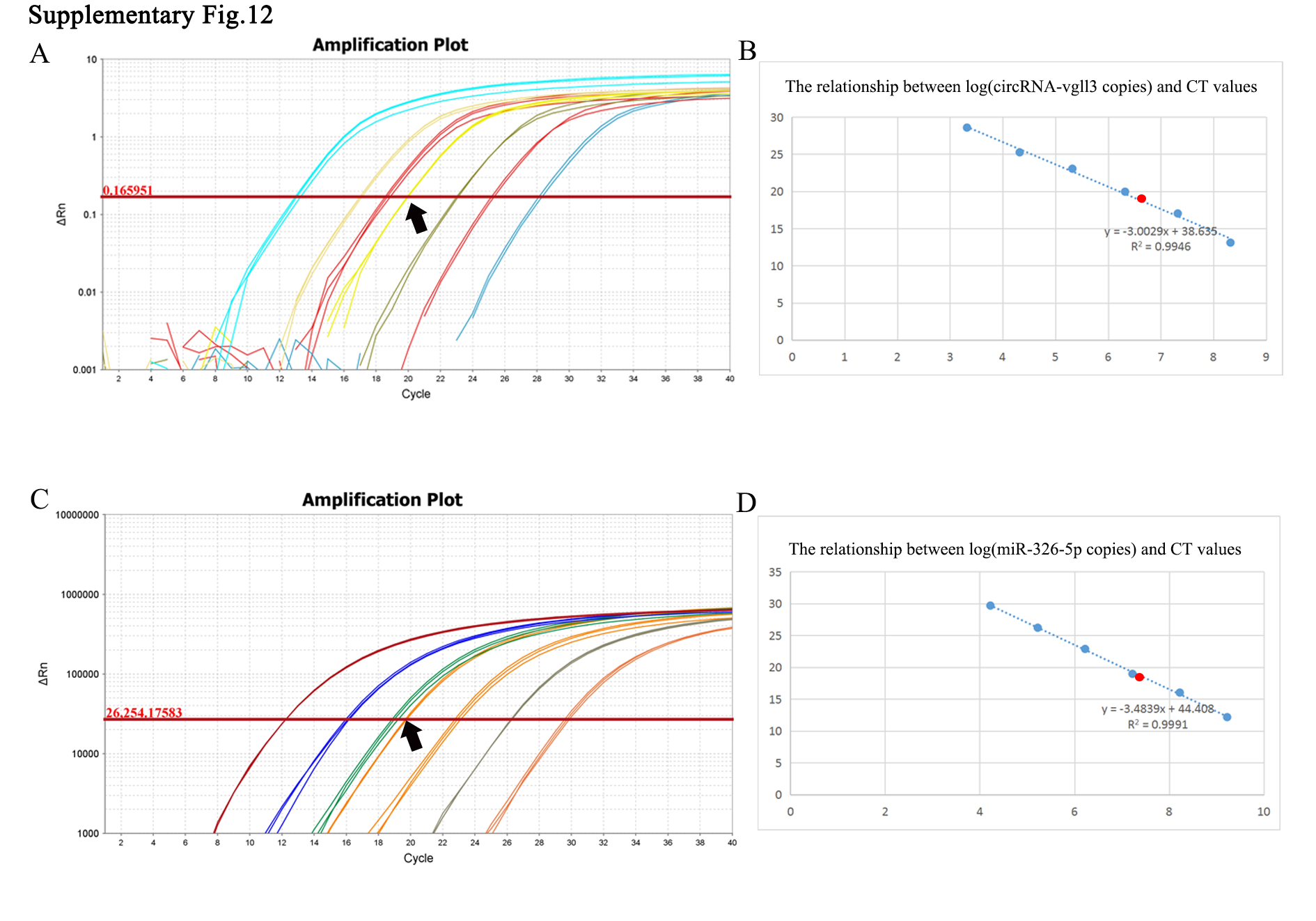

Supplement: Supplementary file 13 — Supplementary Figure 12 [file 41418_2020_600_MOESM13_ESM.tif]

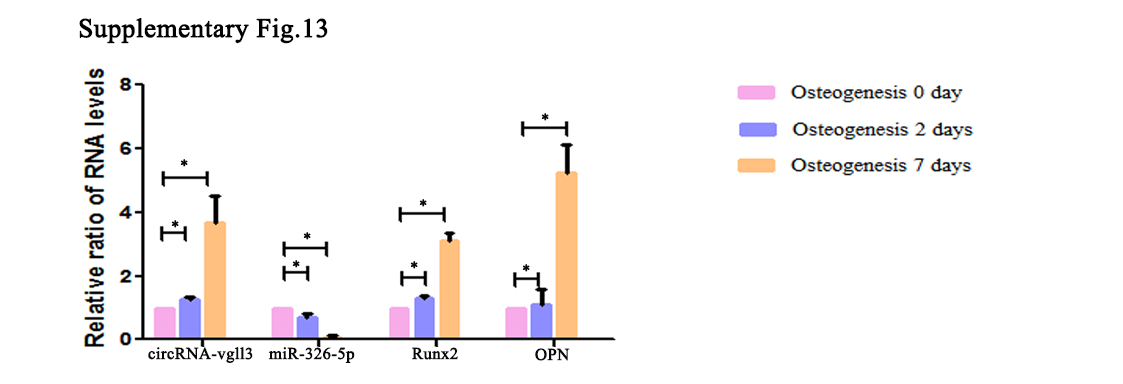

Supplement: Supplementary file 14 — Supplementary Figure 13 [file 41418_2020_600_MOESM14_ESM.tif]
